# 3D calcite heterostructures for dynamic and deformable mineralized matrices

**DOI:** 10.1038/s41467-017-00560-1

**Published:** 2017-09-11

**Authors:** Jaeseok Yi, Yucai Wang, Yuanwen Jiang, Il Woong Jung, Wenjun Liu, Vincent De Andrade, Ruqing Xu, Ramya Parameswaran, Ivo R. Peters, Ralu Divan, Xianghui Xiao, Tao Sun, Youjin Lee, Won Il Park, Bozhi Tian

**Affiliations:** 1 Department of Chemistry, The University of Chicago, Chicago, IL 60637 USA; 2 The James Franck Institute, The University of Chicago, Chicago, IL 60637 USA; 3 The CAS Key Laboratory of Innate Immunity and Chronic Diseases, School of Life Sciences and Medical Center, The University of Science & Technology of China, Hefei, Anhui 230027 China; 4 The Center for Nanoscale Materials, Argonne National Laboratory, Argonne, IL 60439 USA; 5 The Advanced Photon Source, Argonne National Laboratory, Argonne, IL 60439 USA; 6 Biophysics program, The University of Chicago, Chicago, IL 60637 USA; 7 Engineering and The Environment, University of Southampton, Highfield, Southampton SO17 1BJ UK; 8 Division of Materials Science and Engineering, Hanyang University, Seoul, 04763 Korea; 9 The Institute for Biophysical Dynamics, The University of Chicago, Chicago, IL 60637 USA

## Abstract

Scales are rooted in soft tissues, and are regenerated by specialized cells. The realization of dynamic synthetic analogues with inorganic materials has been a significant challenge, because the abiological regeneration sites that could yield deterministic growth behavior are hard to form. Here we overcome this fundamental hurdle by constructing a mutable and deformable array of three-dimensional calcite heterostructures that are partially locked in silicone. Individual calcite crystals exhibit asymmetrical dumbbell shapes and are prepared by a parallel tectonic approach under ambient conditions. The silicone matrix immobilizes the epitaxial nucleation sites through self-templated cavities, which enables symmetry breaking in reaction dynamics and scalable manipulation of the mineral ensembles. With this platform, we devise several mineral-enabled dynamic surfaces and interfaces. For example, we show that the induced growth of minerals yields localized inorganic adhesion for biological tissue and reversible focal encapsulation for sensitive components in flexible electronics.

## Abstract (web summary)

Minerals are rarely explored as building blocks for dynamic inorganic materials. Here, the authors derive inspiration from fish scales to create mutable surfaces based on arrays of calcite crystals, in which one end of each crystal is immobilized in and regenerated from silicone, and the other functional end is left exposed.

## Introduction

The active matrices in artificial dynamic systems frequently rely on stimuli-responsive molecules or polymers, liquid droplets, colloidal particles, cellular components and electronic circuits^1–7^. Surprisingly, naturally occurring minerals, which are part of highly dynamic ecosystems, have not been included in these applications. Calcite can be synthesized under ambient conditions, and it serves as a model system for understanding natural biomineralization^8–15^. However, over the past two decades, most efforts in synthetic calcite have been focused on mechanistic studies of crystal growth and kinetics^8–16^, with sporadic steady-state applications in very few areas (such as optics^15, 17–19^). We hypothesized that the design and implementation of new three-dimensional (3D) architecture in calcite-based building blocks, and subsequent exploration of their unique reaction dynamics (e.g., symmetry breaking) may open up new avenues for inorganic active matrices^1^, and may suggest new pathways for biomimetics and biomineralization^20–22^.

In this work, we demonstrate a new 3D calcite building block in which each structural unit has different growth kinetics and functions. We expand the applications for minerals, and in particular we explore their utilities in flexible electronics and underwater adhesives. Notably, the underwater adhesive explores a novel solid-state localized adhesion mechanism. In addition, we also synthesize a curved, monolithic and minimally strained calcite lattice, with a morphology reminiscent of cocolithophore. All the growth processes, mechanistic studies and application demonstrations are performed under ambient conditions (e.g., at room temperature). Our work suggests a new path for dynamic inorganic systems by enabling regeneration sites.

## Results

### Building block structure and the reaction dynamics

**Fig. 1:**
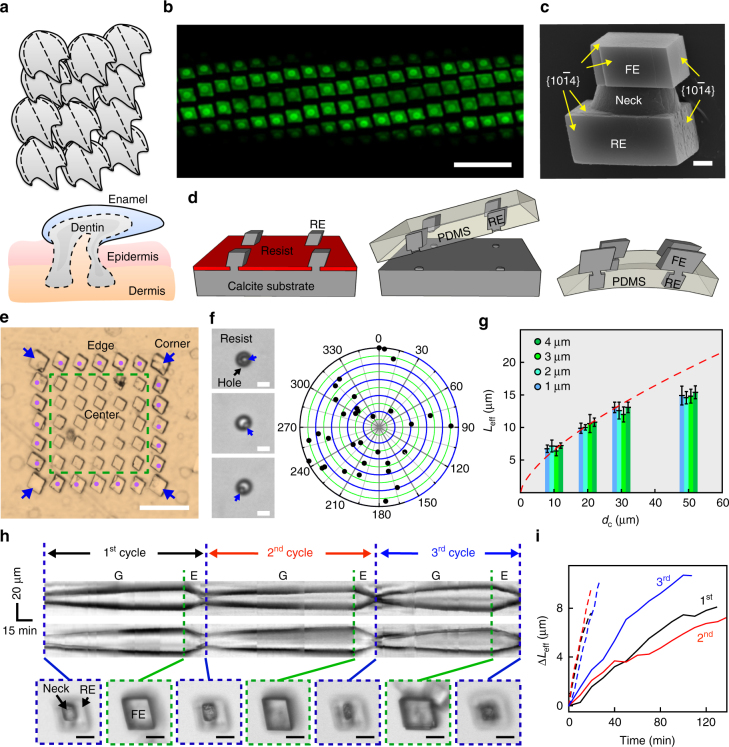
3D calcite building blocks and their growth kinetics. **a** Schematic representation of placoid scales, showing heterojunctions and compartments. The lower portion of dentin is rooted in a pocket of epidermis/dermis. **b** Confocal fluorescence microscope image of a deformable, FITC-BSA-doped calcite matrix. *Scale bar*, 40 μm. **c** SEM image of a calcite heterostructure grown by a tectonic approach, showing an RE, a neck and an FE. *Scale bar*, 1 μm. **d** Schematic representation of mineral matrix fabrication. **e** Optical microscopy image of a 7 × 7 calcite crystal array. *Scale bar*, 50 μm. *Blue arrows*, corner; *magenta dots*, edge; within *green dashed box*, center. **f** Optical microscopy images (*left*) and distribution mapping (*right*) of nucleation in the hole pattern. *Scale bars*, 5 μm. *Blue arrows* mark the crystallites right after nucleation. **g** Neck size-dependent *L*_eff_ − *d*_c_ plot, only center region calcites were considered. *Red dashed line* is the kinetics fitting. *Error bar* indicates standard deviation (*n* = 14). *L*_eff_ is defined in the Supplementary Fig. 7. **h** Kymographs (*upper* and *middle*) showing mutable behaviors of a calcite building block, recorded along cross-sections parallel with the top/down (*upper*) and left/right (*middle*) crystal edges. *Bottom*, snapshots at the transition time points; *scale bars*, 10 μm. ‘G’ and ‘E’ denote growth and etching, respectively. **i** *L*_eff_—reaction time plot for the mutable calcite in **h**. *Solid and dashed lines* denote the growth and etching behaviors, respectively. *Black, red* and *blue* are kinetics during the first, second and third cycles

Here, we use patterned scales from fishes (Fig. 1a) as a natural inspiration^23, 24^ for constructing active matrices based on arrays of 3D calcite crystals (Fig. 1b, Supplementary Figs 1 and 2). Each scale has an inner portion inserted into its own pocket of epidermis/dermis^24^ (Fig. 1a, lower panel), where accumulation of mineral species and regeneration occurs by specialized cells. With this class of hard biomaterials, the outer portions have multiple functions, including soft tissue protection, body temperature regulation and storage of growth ingredients^24^.

Recapitulating the regenerative behavior in abiological systems is challenging, because the cellular pathway for material regeneration cannot be explored. However, epitaxial growth from robust and oriented seeds can be an option because it allows for deterministic or reproducible crystal growth behavior. With this as a guide, we designed an asymmetrical dumbbell-shaped calcite heterostructure (Fig. 1c), with one end stabilized in a silicone matrix and serving as the seed for epitaxial crystallization (i.e., rooted end, RE), and the opposite end exposed for dynamic functions (i.e., functional end, FE) (Supplementary Fig. 2). The hard calcite heterostructure and the soft silicone matrix mimic the scale and the epidermis/dermis components, respectively. We first synthesized REs by parallel epitaxial growth from a single crystalline calcite substrate with a holey polymer resist as the growth mask (Fig. 1d left, and Supplementary Figs 1a, b and 3a), followed by resist removal (Supplementary Fig. 3b–e), polydimethylsiloxane (PDMS) embedding to form self-templated cavities, PDMS release from the thinner neck regions to pick up REs (Fig. 1d, middle, Supplementary Fig. 3f), and secondary growth for FEs (Fig. 1d, right; Supplementary Fig. 3g, h). The last step allowed for tuning of the mineral surface coverage and the incorporation of nanoscopic materials for new applications (Supplementary Figs 2 and 3h). The final individual building block has tectonic motifs (i.e., RE, FE and a neck region, Fig. 1c), containing two {$10\bar 14$}-capped rhombohedra that are characteristic of calcite single crystals. This PDMS-based interface is reminiscent of other architectures demonstrated for either transfer printing^25^ or structured surfaces^23, 26^; however, the inclusion of the thinner neck region helps prevent delamination during device operation (Supplementary Fig. 4). In addition, the starting single crystalline calcite substrate can be used repeatedly and the mineralization can be done under different conditions (Supplementary Fig. 5).

We studied the growth mechanism and kinetics in finite two-dimensional calcite arrays with different neck sizes (i.e., hole diameters in the resist) and center-to-center distances (*d*_c_). We observed location-dependent size differences within calcite crystal arrays (i.e., corner, edge and center, Fig. 1e), suggesting a lateral diffusion-limited growth kinetics (Supplementary Discussion). A distribution map of the nucleation sites (Fig. 1f, right) shows stochastic coordinates within the hole (Fig. 1f, left), suggesting that the immediately adjacent resist layer did not apply chemical or topographical effect over calcite nucleation. In addition, minimal differences in calcite sizes were observed with hole diameters of 1–5 µm in the mask (Fig. 1g and Supplementary Fig. 3a), although different neck sizes can affect the stress distribution when hybrid matrices are deformed (Supplementary Fig. 6). Above 5 µm, concaved centers formed over individual calcite crystals, supporting a lateral diffusion limited growth mechanism (Supplementary Fig. 3a and Supplementary Discussion). The effective lengths of calcite (*L*_eff_, Supplementary Fig. 7) increase with the *d*_c_, which follows (*red dashed line* in Fig. 1g, and Supplementary Discussion).

1 $$L_{{\rm{eff}}}^{\rm{3}} \propto V = \rho d_{\rm{c}}^2,$$

where *V* is the saturated volume after a single step synthesis, *ρ* is the apparent volume density of precursor particles (e.g., amorphous calcium carbonate and vaterite) per area (Fig. 1g). The deviation at large *d*_c_ (e.g., 50 µm) is due to a simultaneous occurrence of randomly nucleated calcite crystals (which also consumes the precursors)^9^. We therefore identified a maximal *d*_c_ ~ 30 μm for achieving a high purity calcite micro-arrays (Supplementary Discussion).

**Fig. 2:**
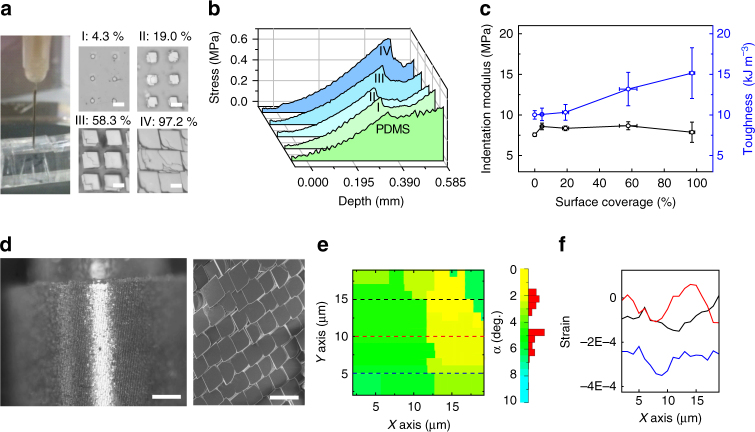
General properties of heterostructured calcite/PDMS matrices. **a** A needle indentation experiment (*left*) for probing the surface mechanics of the hybrid matrices with different mineral coverage (*middle* and *right*). The injection needle (*ϕ*_out_: 0.41 mm, *ϕ*_in_: 0.21 mm) and the hybrid matrix were mounted on a movable upper plate and a stationary bottom stand of the rheometer, respectively. The percent numbers denote calcite surface coverage in four groups of samples (I–IV). *Scale bars*, 10 μm. **b** Indentation curves of calcite/PDMS matrices and **c** corresponding indentation modulus/toughness as a function of surface coverage. *Error bar* indicates standard deviation (*n*_modulus/toughness_ = 5, *n*_coverage_ = 21). **d** Optical micrograph of a curved and fully mineralized lattice (*left*), with individual calcite building blocks merged as highlighted in SEM (*right*). *Scale bars*, 200 μm (*left*) and 40 μm (*right*). **e** Crystal orientation map by X-ray diffraction (*left*), *scale bar* (*right*), and **f** line-scanned strain map of a fully mineralized matrix, from an area primarily covering two merged crystals

**Fig. 3:**
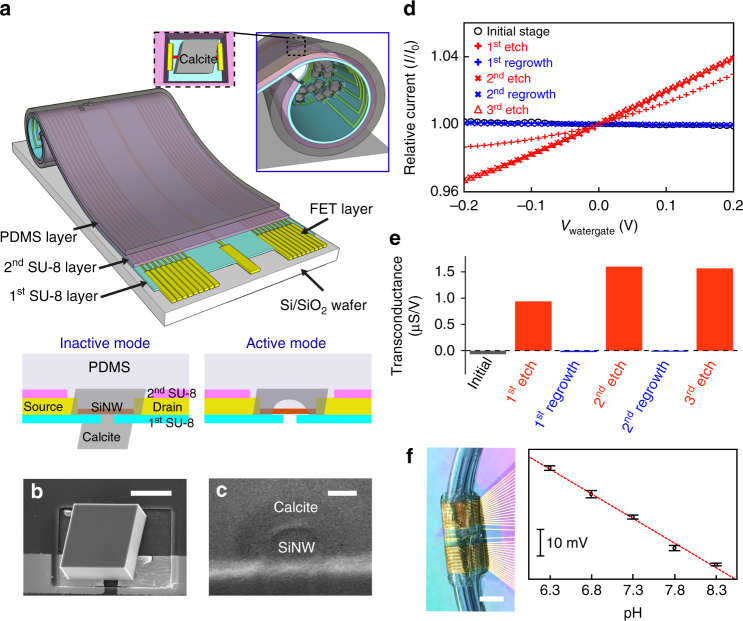
Calcite matrix as focal encapsulation for flexible Si FET. **a** Schematic representation of the tubular construct. Metal interconnects for FETs are passivated with two SU-8 layers, while SiNW can be encapsulated in calcite RE which is inserted in PDMS layer. FE of individual calcite heterostructure is exposed in the lumen for solution access. **b** SEM image of calcite RE-covered FET, recorded before PDMS deposition. *Scale bar*, 20 μm. **c** Cross-section SEM image showing the calcite/SiNW interface, scale bar is 100 nm. **d**, **e** Water-gate characteristics, showing relative drain-source current (normalized to the inactive state current at zero gate bias) vs. water-gate voltage (*I* − *V*_wg_) curves at a drain-source voltage of 0.1 V **d** and transconductances **e** during repeated etching-regrowth cycles. **f** Photograph of a microfluidic SiNW FET construct connected to polycarbonate tubings (*left*), allowing flow of mineralization precursors, etching buffer and solutions for pH sensing experiments. The calculated surface potential change of the Si FET sensor (*right*) vs. solution pH indicates that the response is linear and the slope of the fit (*red dashed line*) yields a response of 22.8 mV/pH. *Scale bar*, 5 mm. *Error bar* indicates standard deviation (*n* = 8)

**Fig. 4:**
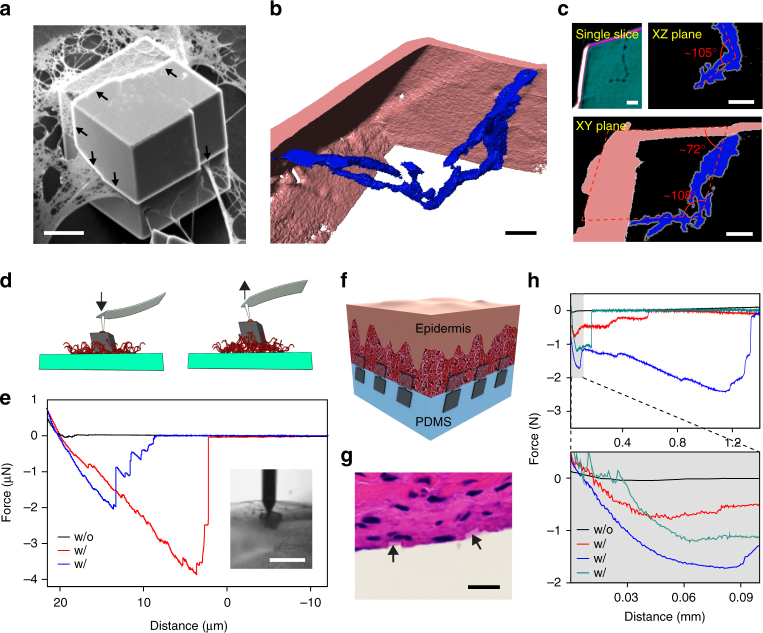
Calcite matrix as inorganic localized adhesions for biological tissues. **a**, **b** A calcite crystal incorporating collagen fibrils. **a** SEM image. *Scale bar*, 10 μm. **b**, **c** 3D reconstructed transmission X-ray microscope image **b**, and a single slice, and the projection images over XZ and XY planes **c**. *Scale bars*, 2 μm. **d**, **e** Schematic representation **d** and representative force-distance recordings **e** of the AFM studies of the interaction between single calcite crystals and collagen coating. (*inset*) Optical micrograph taken during one measurement. The AFM tip with a glue droplet first moved downward until it contacted the top facet of a calcite crystal. The force was only recorded during the retraction of AFM tip/calcite. ‘w/o’ denotes without collagen incorporation, and vice versa. *Scale bar*, 20 μm. **f**–**h** Flexible tissue adhesives with inorganic localized adhesions, demonstrated with a schematic **f**, H&E staining image **g** and force-distance recordings **h**. *Scale bar*, 10 μm

To show the regenerative dynamics, we analyzed the kymographs of a single calcite heterostructure undergoing three cycles of growth and etching (Fig. 1h, Supplementary Fig. 8 and Supplementary Movie 1). Results showed an average lateral growth rate of ~72 nm min^−1^ and an average dissolution rate of ~423 nm min^−1^ (Fig. 1i) under the preparation condition (*d*_c_ = 30 μm, etching pH ~ 5). While FE was responsive to solution changes and showed mutable morphologies, RE remained stable, suggesting a symmetry breaking in the growth kinetics of calcite heterostructures. Stable REs are critical to regeneration of the mineral array, as well as to preventing heterostructure delamination during the dynamic device operations (Figs 2–4).

### Mechanics of the hybrid matrices

Next, we studied the mechanics of the scale/epidermis-like hybrid calcite/PDMS surfaces (Supplementary Fig. 9a, b) with a needle indentation experiment (Fig. 2a). Results show that with increasing calcite surface coverage (up to ~97.2%), despite an enhancement in toughness (up to ~51.3%), the indentation modulus change is minor (< 4.0%) (Fig. 2b, c). Optical images show irregular rupture cracks with an average 13.6% reduction in the crack distance-to-length ratio (Supplementary Fig. 9d), suggesting that the inclusion of calcite crystal arrays deflected the crack propagation in PDMS and toughened the surfaces^27^. The addition of calcite (up to ~97.2%) did not yield plastic deformation before the rupture points **(**Fig. 2b). In addition, finite element simulation suggests that the neck region is likely the crack initiation site (Supplementary Fig. 10 and Supplementary Movie 2). For pure calcite substrate, indentation caused significant crack propagation along {$10\bar 14$} facets, with a crack length up to millimeters (Supplementary Fig. 9c). The demonstrations of deformability in large area (~1.0 cm^2^) calcite arrays (Fig. 2a–c) and responsive morphologies (Fig. 1h, i) in only a portion of the heterostructures suggest new opportunities in mineral materials from synthesis to device applications.

### Monolithic and curved calcite superlattice

We first explored the possibility of synthesizing monolithic and curved calcite superlattice (Fig. 2d–f), a structure reminiscent of naturally occurring calcite exoskeleton (e.g., coccolithophore, Supplementary Fig. 11a); however, it has been a significant challenge to achieve with known synthetic approaches. Leveraging the observed deformability in a heterostructure matrix, we developed a two-step synthesis. Briefly, the hybrid calcite-PDMS surfaces were synthesized and bent at a fixed curvature (0.5–10 mm^−1^), followed by a secondary crystal enlargement and merging to joint adjacent FEs (Supplementary Fig. 11b). The calcite merging was confirmed by the formation of a center-meter scale, freestanding construct (Supplementary Fig. 11c), and the observation of cracking and peeling-off of the mineralized shells upon indentation (Supplementary Fig. 11d). Micro X-ray diffraction (μ-XRD) by X-ray Laue micro-diffraction (Supplementary Fig. 12) confirms bending in the monolithic structure, with a ~3° orientation difference (Fig. 2e, right) between two adjacent calcite crystals (Fig. 2e, left). This angle yields a local curvature of ~0.57 mm^−1^, which corresponds with the global curvature (~0.60 mm^−1^) estimated from the geometry of the macroscopic object (Fig. 2d). Meanwhile, the residual strain, which is measured by μ-XRD (Supplementary Fig. 12a) line scans across the same area (*dashed lines* in Fig. 2e, left), is mostly compressive (likely originated from the crystal merging at the boundary and the bending of PDMS substrate) and is < 0.05% (Fig. 2f).

### Focal encapsulation for flexible electronics

Beyond adjoining adjacent building blocks (Fig. 2d–f), the driving force from continuous crystal growth also suggests that nanoscale objects may be sealed inside a flexible mineralized matrix, especially given recent advances in fibril^16^ and particle^14^ encapsulation within calcite crystals^14, 16^. The development of this idea would yield materials and devices beyond those that simply mimick the structure and mechanics of scales.

To this end, we have configured a flexible silicon nanowire (SiNW) field effect transistor (FET)^28^ array with calcite as a focal encapsulation material for the sensor (Fig. 3a). We chose calcite for this application because the material is biocompatible, rigid (Young’s modulus, ~80 GPa), electrically and thermally insulating (electrical conductivity < 10^−8^ Ω^−1^ cm^−1^; thermal conductivity, ~3.5 ~ 5.6 W m^−1^ K^−1^_,_ at 273 K), and most importantly, can be grown in ambient conditions. We devised this focal encapsulation configuration vs. a traditional global layer because the isolated calcite encapsulation won’t cause large change in the original matrix modulus (Fig. 2a–c). We targeted utilities in flexible electronics because the focal calcite encapsulation can potentially reduce the unintentional chemical, thermal, electrostatic and more importantly, the mechanical damages, to some associated nanoscale building blocks (e.g., Si nanowires). We expect to see such potential applications when devices are not in use but must still be exposed to environmental perturbations (e.g., solution degradation^29^ or unintentional large mechanical deformation to the flexible substrates).

We used multiple steps of growth and transfer to incorporate single ‘plug-like’ calcite heterostructures over individual FET devices (Supplementary Fig. 13). The first growth step through a local orifice introduced the RE and sealed the FET channel (Fig. 3b, c), while the second growth of FE strengthened the sealing and added the solution-responsive dielectrics component for FETs (Fig. 3a). At ‘inactive’ mode (Fig. 3a, lower left), SiNW FET is fully encapsulated from its solution environment. Critically, the focal encapsulation with calcite heterostructure significantly reduces the stress over SiNW upon deformation of the flexible substrate, confirming the mechanical protection (Supplementary Fig. 14 and Supplementary Movie 3). Upon etching of the calcite ‘plug’ (buffer pH ~ 5, ~20 min), FET is at ‘active’ mode (Fig. 3a, lower right) and can be used as a chemical sensor under conditions where one must approximate biological conditions.

Since the FET array was made across flexible substrates (i.e., SU-8 and PDMS, Fig. 3a), we assembled a tubular construct (Fig. 3a and Supplementary Fig. 15) for microfluidics, where solutions with different compositions can be delivered for calcite etching/regrowth and temporary electrical sensing when the FETs were exposed. Representative normalized current vs. water-gate curves and the corresponding transconductances (at zero watergate voltage) indicate that calcite sealing is sufficient to mute the electrical response of a SiNW FET (Fig. 3d, e). In addition, activating the devices from their ‘inactive’ state can be achieved for at least three times without losing REs (i.e., the capability for re-sealing). Upon calcite etching, the device transconductances reached values of 0.9 ~1.6 μS V^−1^, with fluctuations likely due to variable Si/calcite interfaces after each etching. At ‘Active’ mode, a representative tubular SiNW FET device (Fig. 3f) can perform pH sensing (Supplementary Fig. 16) with a linear response (~22.8 mV per pH) and a sensitivity of at least 0.5 units within 6.3 ~ 8.3 pH, a range relevant to pathological and physiological conditions. A similar principle can be extended to modulate graphene-based FET devices (Supplementary Figs 17 and 18).

### Localized inorganic adhesion

Finally, we explored the incorporation of fibrous materials for underwater, localized and inorganic materials based adhesive application. Underwater adhesives^30, 31^ are important for many naval and medical applications, but their options are few because many intermolecular forces that are strong in the gas phase or a vacuum are attenuated significantly in salt solutions. We targeted localized adhesion because many soft and porous materials (e.g., biological tissues or hydrogels) are prone to irreversible damage if global adhesives are applied to their surfaces. Inorganic materials can be a class of unique candidates for achieving localized adhesion given their low mobility/diffusivity in porous medium.

To evaluate this possibility, we first performed single particle study. We synthesized freestanding calcite microcrystals over silica substrate with a thin layer of collagen coating. Scanning electron microscope (SEM) image (Fig. 4a) and transmission X-ray microscope tomography **(**Fig. 4b, c and Supplementary Fig. 12b) showed the incorporation of individual collagen filaments or their interconnected mesh inside calcite single crystals (Supplementary Fig. 19). The orientations of the observed planar defects (Fig. 4a, *arrows*) and the characteristic 105 ~ 108° angles in tomography projections (Fig. 4c) suggest that the incorporation occurs preferentially along {$10\bar 14$} planes. Next, we carried out atomic force microscope (AFM) detachment experiments over individual free-standing calcite crystals with and without collagen incorporation (Fig. 4d, e). The AFM tip was used to pick up and apply a droplet of glue onto the calcite top surfaces, followed by lifting the crystals from the substrate. With collagen incorporation, the representative detachment force and work are 2.04 μN and 11.02 pJ, respectively, while collagen-free calcites yielded values of 1 ~ 2 orders of magnitude less.

We applied this principle to the flexible calcite heterostructure matrices by attaching them to rat skin tissues and allowing for FE growth in a calcium-supplemented phosphate buffered saline solution (Fig. 4f–h and Supplementary Fig. 20). Upon focal mineralization, the energy needed to detach PDMS substrates from skin tissues increased by 2 ~ 3 orders of magnitude (Fig. 4h). Haemotoxylin and eosin staining (Fig. 4g) revealed incorporation of extracellular matrix in spatially separated calcite FEs, suggesting inorganic localized adhesions. This adhesion mechanism is enabled by mineralization over patterned focal points, and we note that permanent adhesion is hard to achieve because calcite can be etched very slowly even under ambient and neutral conditions. Nevertheless, given the mutable property (i.e., etching and regrowth) of the calcite heterostructures and their partial surface coverage over PDMS (i.e., most matrix areas are free of the calcite-based solid adhesive), this hybrid matrix shows promise as far as establishing transient (i.e., temporary adhesions) and minimally invasive adhesions at interfaces. The adhesion strength (~177,300 N m^−2^) and the work of adhesion (~158.7 J m^−2^) are both within the ranges of those recorded from other underwater adhesives (Supplementary Table 1). In both single crystal and ensemble measurements, we have observed stepwise detachment behaviors, with large variations among samples that are likely due to interface heterogeneities.

## Discussion

In this work, we have constructed a mutable and deformable array of mesoscale calcite heterostructures that are partially locked in silicone. With this new platform, we devised several dynamic surfaces and interfaces that are enabled primarily by mineral components. For example, we generated deformable surfaces with tunable toughness (up to ~51.3% increase) while maintaining a similar indentation modulus, and a curved mineralized shell with monolithic and minimally strained (< 0.05%) lattices. We formulated a flexible silicon FET device where a mutable calcite microarray serves as the localized encapsulation material (by which sensing devices can be activated/deactivated on-demand in a microfluidic system). Finally, we established a new mechanism for underwater adhesives, in which the induced growth of minerals serves as an inorganic localized adhesion for biological tissues or other surfaces. Our work provides a new platform for studying crystal growth dynamics (Supplementary Figs 21–27), expands the forms and applications of biomimetic materials^5, 6, 9, 15, 32, 33^, and suggests a scalable pathway toward responsive inorganic systems.

## Methods

### Preparation of flexible calcite-PDMS matrix

$\left\{ {10\bar 14} \right\}$ calcite substrates were prepared from bulk natural calcite crystals by mechanical cleavage. In parallel, 600-nm-thick SiO_2_/Si substrate (NOVA Electronic Materials) was coated with e-beam resist (PMMA C4, MicroChem) or photoresist films (S1813 or S1805, Microchem), and circular hole arrays with diameters of 1, 2, 3, 4, 5, 8, 10, 15 and 20 μm and center-to-center distances of 6, 8, 10, 12, 15, 20, 30 and 50 μm were defined by conventional lithography techniques. The patterned SiO_2_/Si substrate was then immersed in a 1:10 diluted hydrofluoric acid (HF, 49%, Sigma-Aldrich) to remove SiO_2_ film and thereby top polymer film was detached from Si substrate (Supplementary Fig. 1b). The polymer layer was transferred onto deionized (DI). water to rinse residual HF and then mounted onto a pre-cut calcite substrate. The calcite substrate covered with a patterned resist layer was dried under ambient air at room temperature for 5 h. The sample was then immersed into an aqueous calcium chloride solution (CaCl_2_, 10–80 mM, Fisher Scientific) contained in a petri dish. The petri dish and ammonium carbonate ((NH_4_)_2_CO_3_, 5 g, Fisher Scientific) were placed together inside a sealed desiccator. The reaction began with the sublimation of (NH_4_)_2_CO_3_ and subsequent dissolution of CO_2_ into CaCl_2_ solution. Dissolved CO_3_^2−^ ions reacted with Ca^2+^ ions in the solution, yielding precipitation/dissolution of allotrope forms of nanoparticulate calcium carbonates (e.g., amorphous calcium carbonate and vaterite). These calcium carbonates act as precursors to calcite crystal (RE) array grown out from the patterned hole array. After 1 ~12 h, we rinsed the sample surfaces with DI water and removed the resist film by acetone. The sample was then sequentially rinsed with isopropanol and DI water for three times.

Next, base elastomer and curing agent from the Sylgard 184 PDMS kit (Dow corning) were vigorously premixed at a weight ratio of 10:1 for 5 min. The mixture was then applied onto the calcite RE array and left at room temperature for 8 h to remove air bubbles. After further curing at 80 °C for 4–6 h, the PDMS film with the embedded RE array was peeled off from the calcite substrate using a tweezer. Finally, calcite FE array with variable surface coverage were overgrown on the RE array embedded in the free-standing PDMS film under a condition similar to the case of RE growth. After overall growth, the individual calcite crystal has tectonic motifs of FE (outside of PDMS) and RE (inside of PDMS) that were connected by neck segments (Fig. 1c). This procedure can be repeated multiple times from a single calcite substrate with mechanical polishing step included in between (Supplementary Fig. 5).

### Preparation of curved and monolithic calcite-PDMS matrix

A flexible hybrid calcite-PDMS matrix was bent and adhered onto the outer surface of a glass tube (the diameter, *ϕ*, varies, e.g., *ϕ* = 2 − 20 mm), with the individual calcite FEs facing outward. The PDMS was then firmly attached to the tube surface by applying epoxy glue (5 Min Epoxy, Devcon) at both ends of the film. The calcite growth was repeated several times with fresh precursor solutions until each calcite crystal was sufficiently enlarged and eventually merged with adjacent crystals (Supplementary Fig. 11b).

### Measurement of surface mechanics from hybrid calcite/PDMS matrix

The mechanics of the hybrid calcite/PDMS surfaces was tested with a needle indentation experiment. Four matrices with various calcite surface coverage from 4.3 to 97.2% were prepared and their indentation modulus and toughness were measured by a rheometer (Anton Paar Physica MCR 301). The rheometer was equipped with a movable upper plate attached with a 27G1/2 Precision Glide needle (*ϕ*_out_: 0.41 mm, *ϕ*_in_: 0.21 mm, BD Biosciences) and a fixed sample stage at the bottom. Typically, each sample was mounted on the sample stage, and the needle on the upper plate steadily moved downward at a speed of 0.1 mm s^−1^ and eventually pierced the mounted samples while monitoring the stress vs. depth relationship.

### X-ray Laue diffraction microscopy

We examined the orientation and the strain distribution of the curved and monolithic calcite/PDMS matrix by using X-ray Laue diffraction microscopy with submicron spatial and high angular resolution (Beamline 34-ID-E at the Advanced Photon Source, Argonne National Lab, USA). A 7–30 keV polychromatic or monochromatic, focused X-ray beam with a size of 300 nm by 300 nm was achieved by using Kirkpatrick–Baez mirrors. The sample was then mounted at focal point at 45° angle with respect to incident X-ray beam, and the diffracted X-rays from the sample were collected by an X-ray-sensitive area detector (Perkin Elmer flat panel detector) on top of the sample. A series of Laue diffraction images with polychromatic incident X-ray beam at different sample *x*-*y* positions were acquired and indexed for calcite orientation mapping. To determine the strain at each position, Bragg reflection $\left( {10\bar 14} \right)$ was chosen with monochromatic X-rays, and energy scan was performed around Bragg peak for determining lattice spacings of the crystal. All the analysis and calculation were performed using LaueGo software package (https://www1.aps.anl.gov/Science/Scientific-Software/LaueGo) on the Igor pro (6.37, WaveMetrics) platform.

### Preparation of collagen/calcite inclusion and AFM study

Type I collagen fibrils (Corning) were synthesized on a glass cover-slip (Fisher) following the protocol provided by the supplier. The collagen fibril-coated cover slip was washed with DI water for three times, followed by the growth of free-standing calcite crystals using a similar gas diffusion procedure (no REs, FEs or PDMS was used in this case). Finally, the samples were cleaned with DI water and dried in air.

Prior to the AFM (MFP3D, Asylum Research) force recording, the two components of a 30 min epoxy glue (2 Ton Epoxy, Devcon) were intensively mixed and deposited on a glass cover slip. The AFM cantilever (App Nano ACTA) with a tip was lowered until the tip contacted the glue. The cantilever tip was lift up rapidly to pick up a small droplet of glue. With an optical microscope, the AFM tip was positioned over the top of a calcite crystal that incorporated collagen fibrils. The tip was lowered until it touched the calcite and then kept at this position for 2–3 h to ensure firm attachment to the selected calcite. Finally, the cantilever was retracted to a distance of up to ~25 μm and at a speed of 5 μm s^−1^.

### Transmission X-ray microscopy (TXM)

Full-field nano-computed tomography was performed with a transmission X-ray microscope at sector 32-ID of Advanced Photon Source in Argonne National Lab. A calcite crystal containing collagen fibers was first mounted onto a micromanipulator with a focused ion beam system (FEI, USA, Nova 600 NanoLab). The micromanipulator was secured on a custom-built holder for the data collection. Image acquisition was conducted with a monochromatic beam tuned at 8 keV and the condenser and the objective lens. X-rays are focused to the sample by a beam-shaping condenser (BSC), i.e., a mosaic of diffraction gratings organized in concentric rings. The round-shaped BSC can collect a large portion of the beam with its diameter of 1.6 mm. A 180 μm large Fresnel zone plate with 30 nm outer most zone width was used as a microscope objective lens, in order to magnify radiographs of the sample placed on a high-accuracy air-bearing rotary stage. With a charge-coupled device (CCD) -sample distance set to 3.4 m, a magnification of ~47 was obtained. The X-ray detection system corresponds to an assembly comprising a scintillator (LuAG), a 5× microscope objective, a 45° mirror and a low-noise fast CCD cooled at −40 °C. The voxel width obtained in this geometry was 27.6 nm and the field of view was ~70 × 60 μm^2^ while the illumination beam coming from the BSC has a disk shape with a diameter slightly larger than 70 μm. The true spatial resolution given by the zone plate is ~30 nm. 3D reconstructions were performed with the software Tomopy (http://www.aps.anl.gov/tomopy/), an open source Python-based toolbox for the analysis of synchrotron tomographic data. The 3D iso-intensity surfaces were constructed and visualized using Amira 5.5 (FEI Visualization Sciences Group) and NIH imageJ. Manual segmentation of collagen fibers was carried out based on intensity using a magic wand tool.

### Preparation and measurement of Si FET with mutable calcite plugs

First, a 100-nm-thick Ni layer was thermally evaporated (ATC-ORION-8E UHV, AJA International, Inc.) onto a 4 inch Si substrate coated with a 600-nm-thick SiO_2_ layer. The 2-μm-thick rectangular shape SU-8 (SU-8 2002, MicroChem) islands with central circular holes were fabricated on the Ni layer with photolithography. N-type Si nanowires were synthesized via a gold nanoparticle catalyzed vapor-liquid-solid growth technique in a chemical vapor deposition system, with flow rates of 50 sccm H_2_, 1 sccm SiH_4_ and 1 sccm PH_3_ (1000 p.p.m. in H_2_) at 470 °C for 1 h. As-grown Si nanowires were transferred and aligned on the SU-8 islands by contact printing technique^34^. Following a second lithography, a 2 μm SU-8 supporting layer were defined on the substrate, which was aligned with and connected to the holey SU-8 islands made in the first step. Next, LOR 8A undercut and S1813 photoresist layers were patterned by photolithography, followed by evaporation of 20 nm Cr and 80 nm Au for contact electrodes to Si nanowires. For contact passivation, additional 2 μm SU-8 layer was deposited. Finally, half of the SU-8 layer embedded with Si nanowire devices was released from the substrate by etching the Ni film in 10% HCl solution.

For calcite RE growth, a pre-cut calcite substrate was gently placed between the floating SU-8 layer with Si nanowire devices in water and the SiO_2_/Si substrate. The sample was then slowly lifted up to mount the SU-8 layer onto the calcite substrate, and was dried in air for 5 h to obtain a tight adhesion of the SU-8 layer to the calcite surface. We then placed the sample in precursor solution to grow calcite crystals over the Si nanowire devices through the holes defined in the second SU-8 layer from the calcite substrate. Next, the top surface of SU-8 layer was coated with PDMS layer and cured at 80 °C for 4–6 h. In a final step, the hybrid device-SU-8/PDMS matrix was gently detached from the calcite crystal by razor blade and tweezer. The hybrid matrix could be rolled-up into a tubular form, and be connected to a syringe pump through transparent polycarbonate tubing.

Electrical measurement was carried out using a Keithley 2636A dual-channel source meter with control by custom programmed software (LabView, National Instrument). The drain currents vs. water-gate voltage characteristics of the devices were measured at a drain voltage of 0.1 V and wager-gate voltage in the range of −0.2 to 0.2 V.

### Skin sample preparation and mechanical testing

Skin patches were cut and peeled off from the flanks of P1-P3 Sprague-Dawley neonatal rat pups obtained from Charles River Laboratories International, Inc., and they were cleaned in a phosphate-buffered saline (PBS) solution to remove residual blood. All animal protocols used in this work were approved by the University of Chicago Animal Care and Use Committee.

To integrate the skin sample with the calcite/PDMS matrix, the skin patch was transferred onto a top surface of calcite/PDMS matrix, and was pressed gently to ensure that the inner side of the skin had robust contact with the exposed necks of the calcite crystals. To initiate the growth of calcite FEs, the skin-attached sample was immersed in a 20 mM CaCl_2_-supplemented 1× PBS solution placed inside a sealed desiccator in the presence of (NH_4_)_2_CO_3_ powder for 2 h. After calcite growth, the sample was washed with DI water several times.

Hematoxylin and eosin (H&E) staining was used to visualize the biointerface between the rat skin and the calcite array. The skin tissues were processed with Tissue-Tek VIP 6 Vacuum Infiltration Processor (Sakura Finetek) using standard program (1 h per solution) and then sectioned with a microtome (HM 315 Microtome, Microm). H&E stain was performed in Tissue-Tek® Prisma® Automated Slide Stainers (Sakura Finetek) and the stained slides were clipped in Tissue-Tek® Glas™ g2 Automated Coverslipper (Sakura Finetek).To measure change in adhesive forces involving the regrown calcite, we prepared skin samples with and without calcite regrowth. The skin tissue part and the PDMS part were fixated onto the top and the bottom plate of a rheometer (Anton Paar Physica MCR 301), respectively. The top plate was raised upward at a speed of 10 μm s^−1^ until the skin tissue part was fully detached from the PDMS part fixed to the bottom plate while monitoring the force-distance signal.

### Data availability

Source data for a few figures are available in figshare at https://figshare.com/s/c374f0966d9baa898ae1. The authors declare that other data supporting the findings of this study are available within the article and its Supplementary Information. Additional information is available upon request from the corresponding authors.

## Electronic supplementary material

Supplementary Information

Supplementary Movie 1

Supplementary Movie 2

Supplementary Movie 3
