# Supplementary material for: 3D calcite heterostructures for dynamic and deformable mineralized matrices
Source: Nat Commun. 2017 Sep 11;8:509. doi: 10.1038/s41467-017-00560-1 (PMC5593869; doi:10.1038/s41467-017-00560-1)
Supplement: Supplementary file 1 — Supplementary Information [file 41467_2017_560_MOESM1_ESM.pdf]

Type of file: pdf

Title of file for HTML: Supplementary Information

Description: Supplementary Discussion, Supplementary Figures, Supplementary Table, and Supplementary References

----

Type of file: avi

Title of file for HTML: Supplementary Movie 1

Description: Mutable behaviors of a single calcite heterostructure under three cycles of growth and etching.

Type of file: avi

Title of file for HTML: Supplementary Movie 2

Description: Indentation simulations of the calcite/PDMS matrix. The dynamics of von Mises stress at FE, neck, RE and PDMS matrix were mapped and plotted

Type of file: avi

Title of file for HTML: Supplementary Movie 3

Description: Bending simulations of a Si nanowire with and without a calcite focal encapsulation. The dynamics of von Mises stress along the Si nanowire in both cases were recorded and plotted.

Supplementary Discussion

General growth process for calcite crystals

The growth of calcite crystals follows:

$$Ca^{2+}(aq) + CO_3^{2-}(aq) \xrightarrow{precipitation} CaCO_3(s) \quad (1)$$

where Ca^{2+} ions come from $CaCl_2$ salt, and CO_3^{2-} ions were supplied by the following reactions:

$$(NH_4)_2CO_3(s) \xrightarrow{sublimation} 2NH_3(g) + CO_2(g) + H_2O\ (g) \quad (2)$$

$$CO_2(g) + H_2O\ (l) \xrightarrow{dissolution} CO_3^{2-}(aq) + 2H^+(aq) \quad (3)$$

The precursors can yield various polymorphs of calcium carbonate, *i.e.* hydrated amorphous calcium carbonate (ACC), anhydrous ACC, vaterite, aragonite, and calcite, *etc.* In this discussion, hydrated ACC and anhydrous ACC are treated the same since they have similar characteristics, and aragonite is excluded from consideration given its relatively high crystallization temperature[1]. Considering enthalpies of formation ($\Delta H_{\text{ACC to calcite}}$ = -21.5 ~ -17.6 kJ/mol, $\Delta H_{\text{vaterite to calcite}}$ = -3.4 kJ/mol)[2] and solubilities of calcium carbonate allotropes ($K_{sp,ACC} = 10^{-6.30}$, $K_{sp,vaterite} = 10^{-7.91}$, $K_{sp,calcite} = 10^{-8.49}$)[3,4], it is generally accepted that the crystallization of calcite occurs via nanoparticulate ACC precursors as passing through three phases: ACC → vaterite → calcite (Specifically, (i) initial deposition of ACC nanoparticles; (ii) dehydration of ACC and crystallization to vaterite; and (iii) transformation of vaterite to calcite via a dissolution and reprecipitation mechanism)[5]. Depending on the conditions, a direct transformation from ACC to calcite may also be possible[2].

Growth kinetics of calcite crystals on patterned substrates

In order to elucidate the growth behavior of calcite crystals as well as to improve the controllability, we performed experiments with patterned hole arrays in the resist layers.

Briefly, we covered a single crystalline calcite substrate with a resist layer that was fabricated and isolated from a Si/SiO_2 wafer. The resist contains 7×7 square arrays of circular holes with different diameters (d_h = 1, 2, 3, 4, 5, 8, 10, 15, and 20 μm) and center-to-center distance (d_c = 10, 20, 30, and 50 μm) (**Supplementary Fig. 21**). While the resist layer physically suppresses the nucleation of calcite, the exposed calcite surfaces via the holes served as the nucleation sites for the epitaxial calcite growth, producing micro-patterned arrays of rhombohedral calcite crystals. The growths from these smaller and well-controlled arrays (*vs.* 2D arrays with infinite sizes) allowed the exploration of growth mechanism and kinetics that are equally applicable over the large area matrix. Specifically, we have studied the effect of d_h and d_c on the effective size (L_{eff}) of calcite crystals (L_{eff} is defined in the **Supplementary Fig. 7**).

We observed several unique features in the experiments: (i) the L_{eff} of calcite crystals grown out from the holes (**Fig. 1d**, left) is dependent on their locations within the arrays (*i.e.*, L_{eff} (corners) > L_{eff} (edges) > L_{eff} (centers), **Fig. 1e**); (ii) the location dependent L_{eff} difference generally decreases as d_c increases (*e.g.*, corner/center difference: 86 % and 23 % for d_c = 10 μm and d_c = 50 μm, respectively, **Supplementary Fig. 22**); (iii) minimal differences in L_{eff} were observed with hole diameters of 1–4 μm in the mask (**Fig. 1g** and **Supplementary Fig. 23**), while above 5 μm, concaved centers formed over individual calcite crystals (**Supplementary Fig. 3a**).

Considering all these observations into account, we formulated a hypothesis that the crystal growth is mainly governed by a lateral diffusion-limited kinetics. To justify our hypothesis, we have constructed an analytical model for the calcite growth kinetics based on the following three assumptions (**Supplementary Figs. 24** and **25**).

(1) The total volume of calcite crystals after the growth is proportional to the total amount of initial precipitate (i.e., precursors for calcite) from the supersaturated solutions.

(2) The existence of preferential nucleation sites in the exposed areas suppresses the nucleation of free-standing calcite crystals on the resist surface unless the d_c is large.

(3) We consider two main routes for precursor supply: one is the vertical mass transport from the bulk solution, and the other is the lateral diffusion near the sample surface.

At the early stage, nanoparticulate ACC and vaterite are generated and dispersed in the bulk solution, and some of those particles settle down over the sample surface. Once nucleation from the exposed calcite sites initiates, the pre-deposited ACC and vaterite particles are dissolved and the resultant precursor ions are diffused to nearest calcite crystals and consumed for calcite growth. The ion concentration in the bulk solution remains largely steady and the mass transport along vertical direction is limited. The overgrowth of calcite via the holes is therefore driven mainly by the lateral diffusion of precursors that are released from ACC/vaterite in the immediately adjacent areas (**Supplementary Fig. 24**). Considering mass conservation, the relationship between volume of calcite crystal (V) and supply area (A) can be followed as

$$V = \rho A = \rho \pi R_t^2 \tag{4}$$

where ρ reflects the volume density of precursor particles (*e.g.*, ACC and vaterite) per area, which is dependent on the degree of supersaturation, container geometry (*e.g.*, depth) and the orientation of the growth substrate. R_t is the radius of the circular area in which precursor particles are fully dissolved at the time t. In our experimental system, the volume expension of calcite was limited when the R_t exceeds $d_\mathrm{c}/2$ due to sharing of precursor supply with neighbouring crystals. The maximum V becomes,

$$V_\mathrm{max} = \rho A = \rho d_\mathrm{c}^2 \tag{5}$$

Since V is proportional to L_eff^3, we then have:

$$\alpha L_\mathrm{eff}^3 \cdot \sin^2(\theta) = V = \rho A = \begin{cases} \rho \pi R_t^2 \ (when\ R_t < d_\mathrm{c}/2) \\ \rho d_\mathrm{c}^2 \ \ (maximum\ volume) \end{cases} \tag{6}$$

where α is the (height/effective width) ratio for individual calcite crystal, and θ is the angle between two edges (~ 74.55°). This relation predicts that the maximum L_{eff} (*i.e.*, the effective length one can achieve after a single growth cycle is complete.) is linearly proportional to $(d_{\text{c}})^{2/3}$ and also, volume limitation in ideal case of our experiemntal system. Indeed, this result agrees well the experimental data (**Fig. 1g** and **Supplementary Fig. 23**, when d_{c} < 30 μm).

Additionally, this lateral diffusion-limited crystal growth model can also explain location-dependent crystal size differences. Crystals with smaller 'coordination' number (i.e. crystals at the edge and corner) have extra exclusive area to collect precursors, and consequently, crystal size is bigger than that from the center area (**Supplementary Figs. 22** and **26**).

To evaluate the calcite growth dynamics, we first calculated the 2D diffusion of precursors at each time frame based on Fick's second law[6],

$$\frac{\partial C}{\partial t} = \frac{1}{r}\frac{\partial}{\partial r}\left(r\frac{\partial C}{\partial r}\right) \tag{7}$$

where C is the ion concentration, r is the distance. We considered two boundary conditions as follows:

$$C(R_t) = C_{\text{p}},\ \ C(R_0) = C_0 \tag{8}$$

where C_{p} is the apparent solubility of precursor precipitate, R_0 is the distance from center to the surface of calcite crystal and which is ~ $L_{\text{eff}}/2$, and C_0 is the concentration at surface. Thus, the concentration of precursor will be

$$C = C(r) = \iint \frac{1}{r}\frac{\partial}{\partial r}\left(r\frac{\partial C}{\partial r}\right) = C_0 + \frac{C_{\text{p}} - C_0}{ln(R_t/R_0)} ln(r/R_0) \tag{9}$$

Under steady-state conditions, diffusion flux can be solved by Fick's first law,

$$J(r) = -D\frac{dC(r)}{dr} = -D\left\{\frac{C_{\text{p}} - C_0}{\ln(R_t/R_0)} \cdot \frac{1}{r}\right\} \tag{10}$$

where D is the diffusion constant. In ideal case, a total amount of diffusion fluxes at boundary of adjacent area ($J(R_t)$ should be same as growth rate of calcite crystal (dV/dt),

$$\frac{dV}{dt} = |J(R_t)| \cdot 2\pi R_t \tag{11}$$

Plugging the diffusion flux (10) into this equation, we obtain

$$\frac{dV}{dt} = 2\pi D\left(\frac{C_\mathrm{p} - C_0}{\ln(R_t/R_0)}\right) \tag{12}$$

From the definition of R_0 ($R_0 \sim L_\mathrm{eff}/2$) and equation (3) ($L_\mathrm{eff} = \sqrt[3]{\rho\pi R_t^2/\alpha\sin^2(\theta)}$), growth rate can be derived as

$$\frac{dV}{dt} = \frac{2\pi D\left(C_\mathrm{p} - C_0\right)}{\ln\left(\frac{2}{\sqrt[3]{\rho\pi/\alpha\sin^2(\theta)}} \cdot R_t^{1/3}\right)} = \frac{6\pi D\left(C_\mathrm{p} - C_0\right)}{\ln\left(\frac{8\alpha\sin^2(\theta)}{\rho\pi} \cdot R_t\right)} = \frac{K}{\ln(kR_t)} \tag{13}$$

where K and k are the constant ($K = 6\pi D\left(C_\mathrm{p} - C_0\right)$, $k = 8\alpha\sin^2(\theta)/\rho\pi$). Given that R_t is always bigger than R_0 (thus $R_t/R_0 > 1$), the growth rate of calcite crystal follows a $1/\ln(kR_t)$ curve ($kR_t > 1$). It is implying that the growth rate will be decreased as increase of R_t (**Supplementary Fig. 27)**. In addition, the formation of a neck (*i.e.* after filling of a hole in the mask) should precede the lateral overgrowth of rhombohedral calcite crystal (RE, **Supplementary Fig. 3b**) and as a result, R_t is at least bigger than $d_\mathrm{h}/2$.

Although this model well explains growth behavior of calcite crystal in our system, there are some limitations. For example, we did not consider (i) the nucleation or growth of free-standing calcite crystal, which would occur with large d_c (**Fig. 1g** and **Supplementary Fig. 23**, when d_c > 30 μm), and (ii) the exact volume of neck.

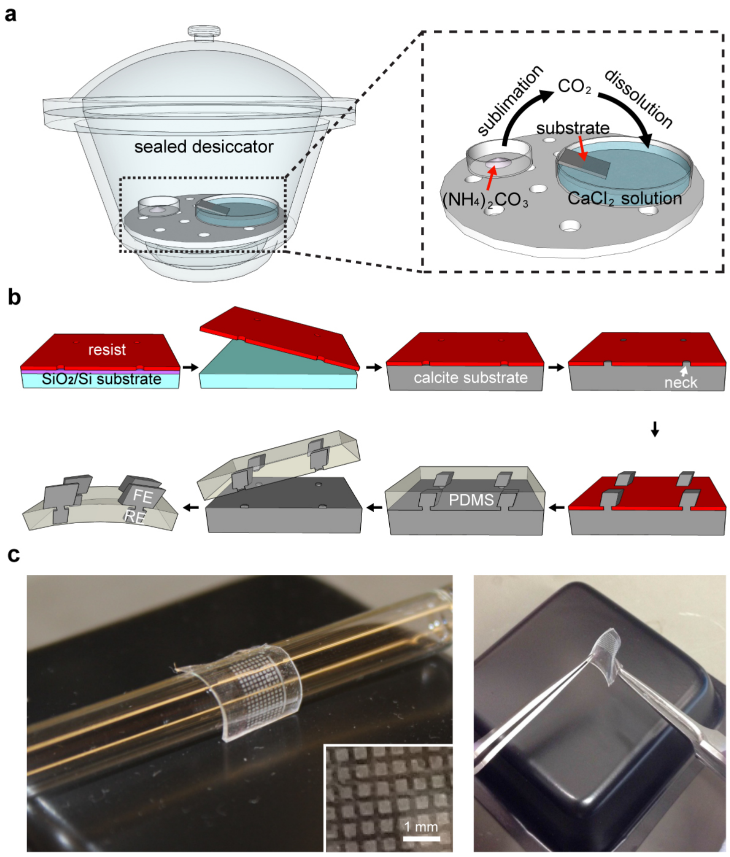

Supplementary Figure 1 | Material synthesis and fabrication of calcite/PDMS matrix. a, Schematic of the setup for calcite growth. $(NH_4)_2CO_3$ powder is sublimated to yield CO_2 and NH_3 gases. The gases are dissolved in $CaCl_2$ solution, which initiates calcite crystallization. **b**, Schematic illustrating the fabrication steps for calcite/PDMS matrix. **c**, Photographs of calcite-silicone hybrid matrixes in a supported (left) and a clamped configuration (right). Inset, zoom-in image highlighting the patterned arrays. A total of up to 405,000 calcite sites are available in this matrix.

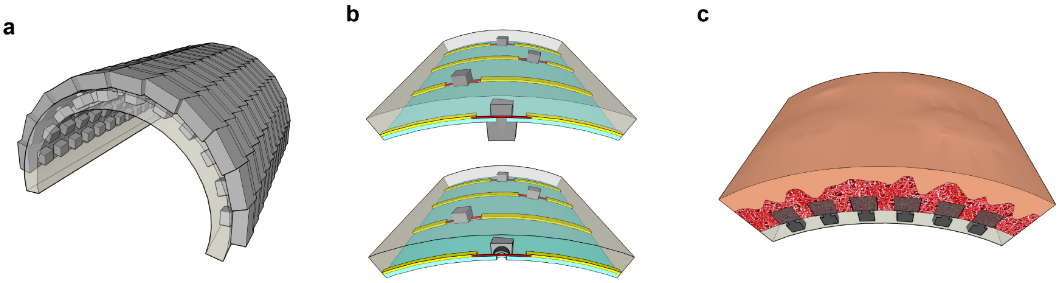

Supplementary Figure 2 | Schematics of various systems based on dynamic and deformable mineralized matrices. **a**, A monolithic fully mineralized 'exoskeleton'. **b**, Responsive calcite 'plug' as reversible and focal encapsulation material for flexible FET sensor, with 'inactive' (upper) and 'active' (lower) modes. **c**, Flexible calcite matrix as solid-state focal adhesions.

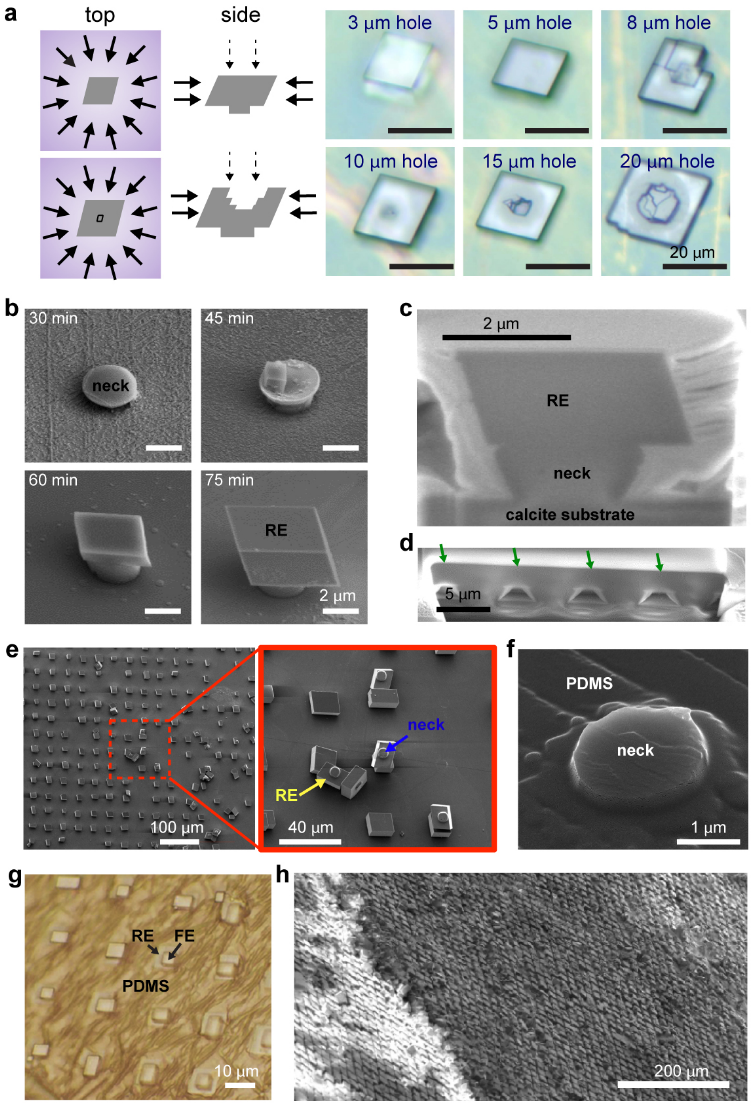

Supplementary Figure 3 | Components for calcite micro-arrays. a, Left: schemes of precursor flux and resulting morphology in growths through resist layers with small (upper left) and large (lower left) hole sizes. Right: optical micrographs of as-grown calcite crystals, with the numbers showing different hole sizes. **b**, SEM images showing the evolution of RE. **c** and **d**, Cross-sectional SEM images of (**c**) as-synthesized RE and (**d**) merged RE array. Samples were prepared by focused ion beam cross sectioning. The green arrows in (**d**) mark the locations for merging. **e**, SEM images of RE/neck array after resist removal. The substrate was scratched to detach and reverse many of the heterostructures. **f**, SEM image showing an exposed neck for subsequent growth of FE, with the RE embedded in PDMS. **g**, Optical micrograph of the hybrid matrix. FEs were made intentionally small for visualizing the PDMS embedded REs. **h**, SEM image of a fully mineralized folded matrix.

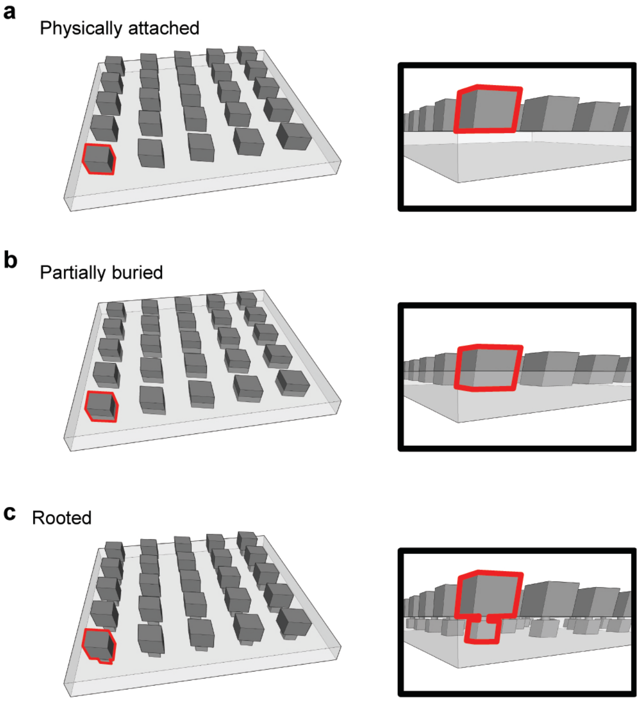

Supplementary Figure 4 | Three possible scenarios of calcite/PDMS hybrid matrices. a, Calcite crystal array is physically attached to PDMS upper surface. In this configuration, calcite crystals could be easily detached. **b**, Part of calcite crystals is buried in PDMS matrix. Calcite crystals are not tightly immobilized in this configuration either. Both configurations **a** and **b** can be derived directly from existing calcite-based building blocks. **c**, Calcite crystals are locked in the PDMS matrix by the Rooted Ends (REs). This configuration is used throughout our work.

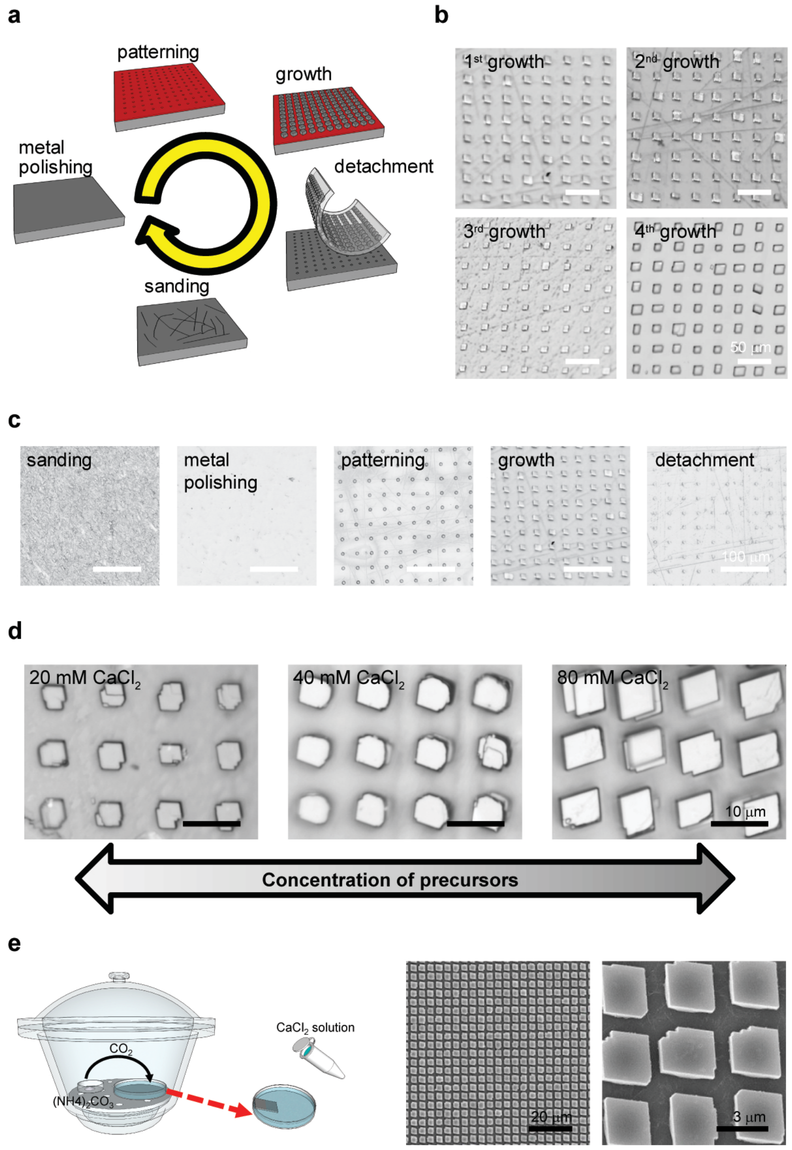

Supplementary Figure 5 | Substrate recyclability and broad synthetic range. a-c, The single crystalline calcite substrate can be recycled for multiple growths of calcite micro-arrays. **a**, A schematic showing the procedure, with sanding and polishing steps. **b**, Optical microscope images of the as-synthesized RE arrays. The 1st growth was done on a freshly-cut calcite substrate. The following growths were done on the same calcite substrate after polishing (2nd to 4th growths). **c**, Optical microscope images showing individual steps of a single cycle. **d** and **e**, Growth of calcite micro-crystals can be achieved in a broad range of conditions. **d**, Optical microscope images of as-grown REs under different $CaCl_2$ concentrations. **e**, Rapid growth of calcite was achieved by mixing $CaCl_2$ and CO_2 pre-saturated solutions. Schematic diagram of a setup for rapid calcite growth (left) and SEM images of as-grown calcite crystal array (right).

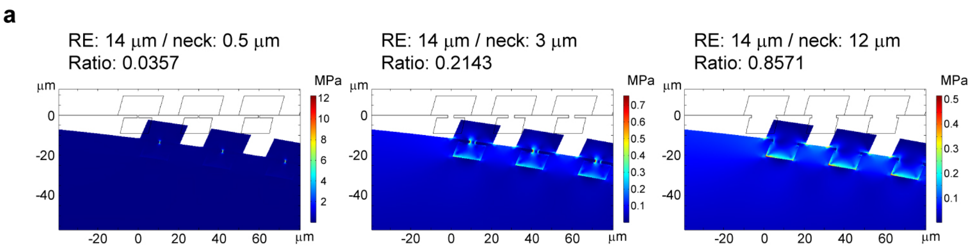

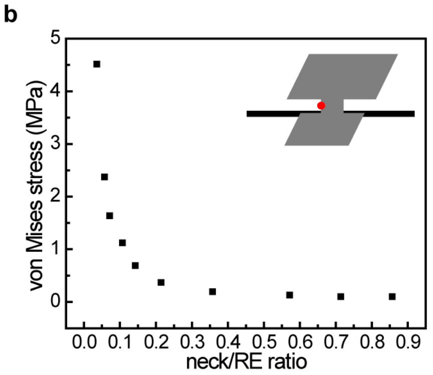

neck (μm)	neck/RE ratio	stress (MPa)
0.5	0.035714	4.516057
0.8	0.057143	2.373319
1	0.071429	1.635764
1.5	0.107143	1.123995
2	0.142857	0.690759
3	0.214286	0.370336
5	0.357143	0.194707
8	0.571429	0.130415
10	0.714286	0.101371
12	0.857143	0.101153

Supplementary Figure 6 | Finite element simulations of the stress distribution in the calcite/PDMS matrix with different neck/RE ratios. **a**, Images of von Mises stress distribution when the calcite/PDMS matrix was bent. When the neck/RE ratio is very small (left), most of the stress is confined to the neck, suggesting a risk of neck failure. When the ratio is large (right), the stress occurs evenly along the bottom RE/PDMS interface, suggesting a risk of delamination of the whole calcite structures. We chose the middle configuration for most of our studies, because the stress is distributed over multiple locations of the calcite heterostructures, which favors a secure anchorage. **b**, A plot of the von Mises stress (on a neck, marked with a red dot) versus the neck/RE ratio, and the tabulated numbers (right). As neck/RE ratio increases, the stress on the neck quickly drops and then reaches a minimum.

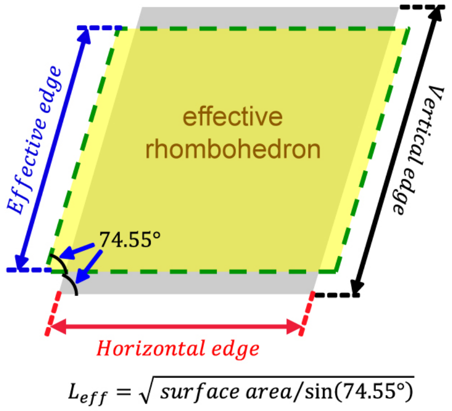

Supplementary Figure 7 | Definition of multiple edges in a calcite crystal. In particular, effective edge is the edge of an ideal and hypothetical rhombohedron which has identical top surface area of a real crystal.

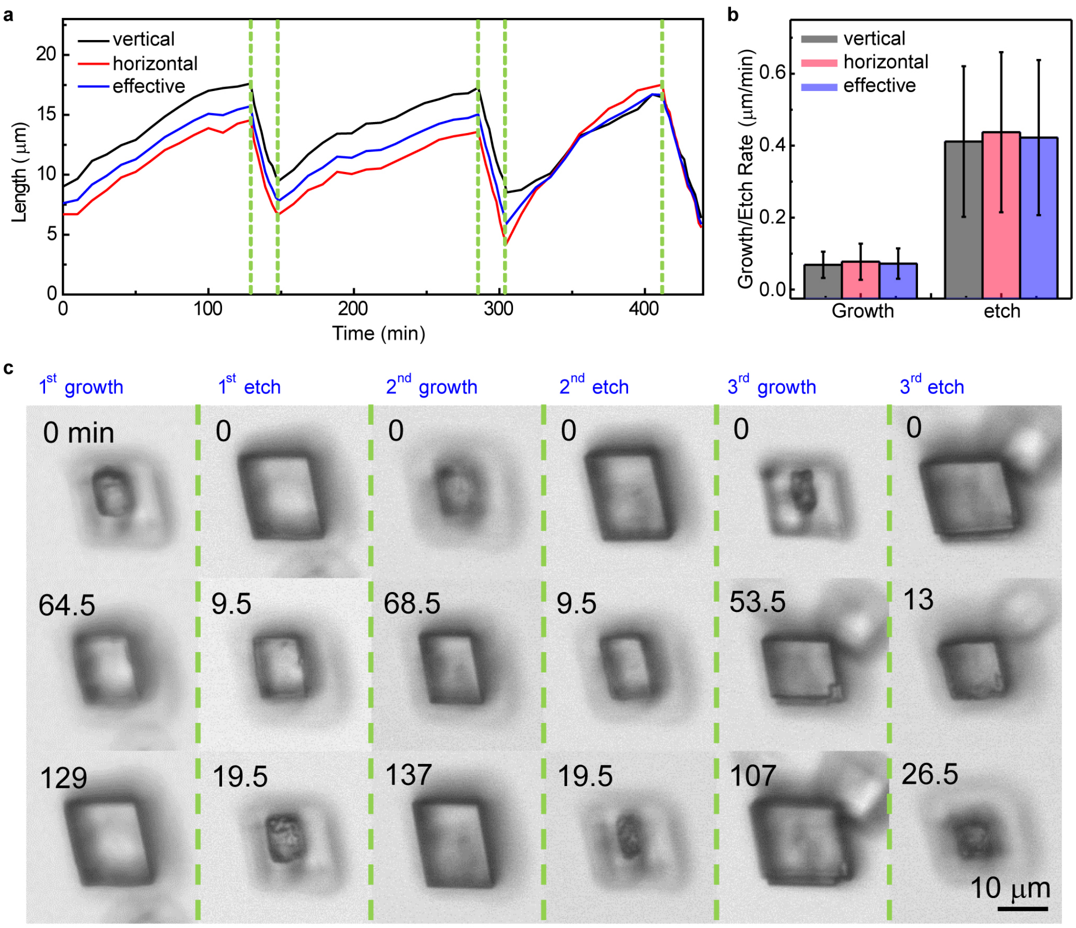

Supplementary Figure 8 | Mutable morphologies. Three cycles of growth and etching of a calcite heterostructure, supporting the data in **Fig. 1h**. **a**, edge length kinetics **b**, growth/etch rate, mean ± 1 SD. n = 3. and **(c)** optical micrograph snapshots at marked time points.

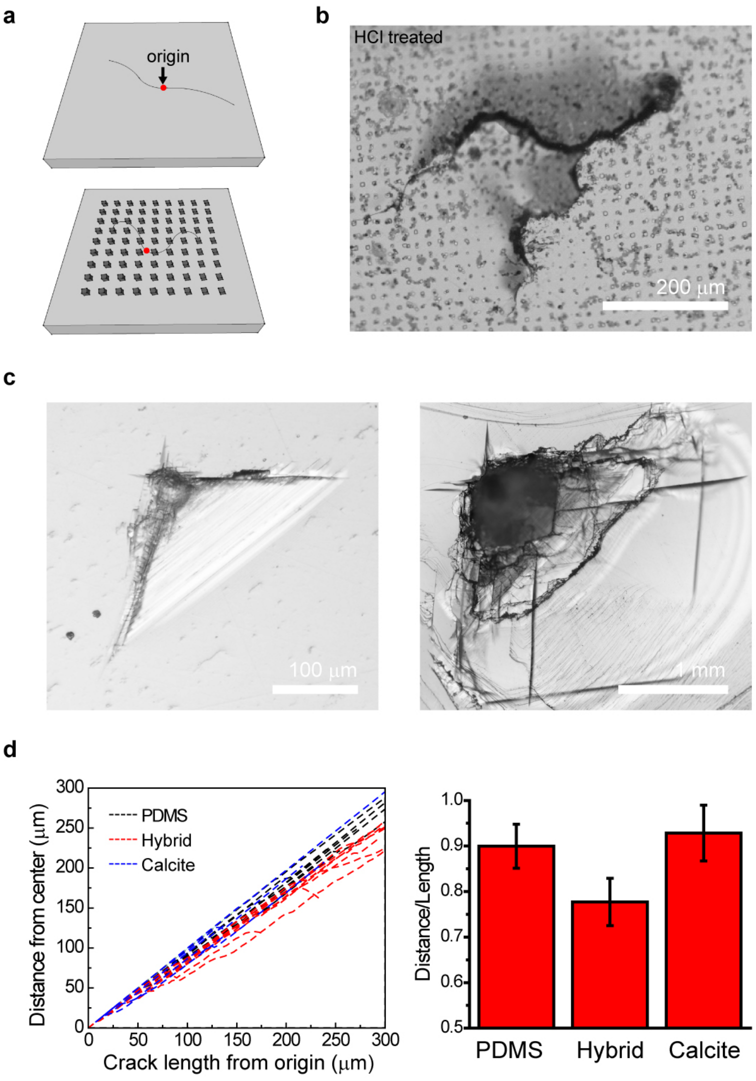

Supplementary Figure 9 | Mineralized sheets. a, Scheme of crack propagations on a clean PDMS surface (upper) and a mineralized PDMS surface (lower). **b**, Optical micrograph of cracks left on a mineralized matrix with original calcite surface coverage of 97.2 %. The calcites were partially removed by HCl etching for better visualization of cracks. **c**, Typical optical micrographs of a bulk calcite substrate upon indentation, showing both smaller (left) and larger (right) cracks. **d**, Distance versus length plot for individual cracks (left) and their ratios (right). If cracks are more linear, a ratio of ~ 1 should be expected. Smaller Distance/Length ratios suggest deflection of cracks in the material. Error bars are one standard deviation from the means (n = 6).

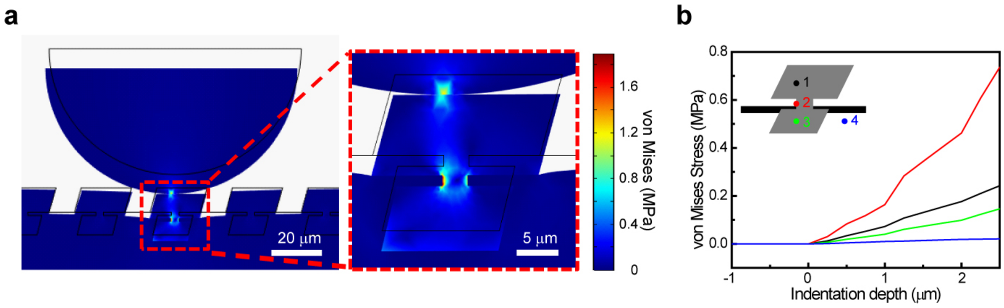

Supplementary Figure 10 | Finite element simulations of a calcite/PDMS matrix upon indentation. a, Images of von Mises stress show that most stress is concentrated at the neck, followed by the contact point. **b**, A plot showing the stress dynamics during the indentation, recorded in 4 locations.

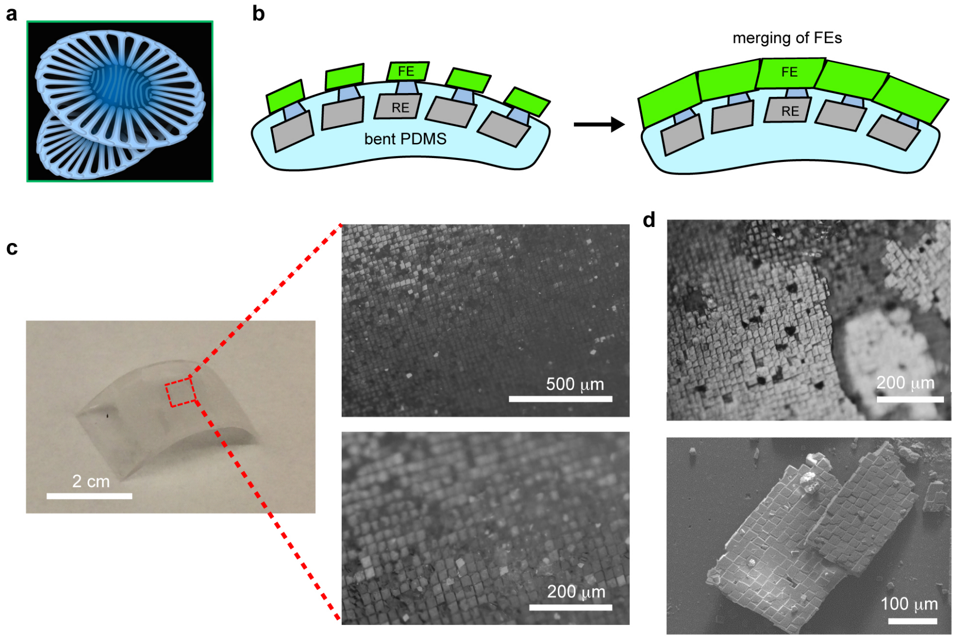

Supplementary Figure 11 | Freestanding and curved calcite/PDMS matrix. a and **b**, Material tectonics. Schematics of *Emiliania huxleyi* coccoliths (**a**) and the formation of a curved and monolithic calcite array (**b**); both structures are made of multiple segments or units. **c**, A photograph (left) and optical micrographs (right column) of a fully mineralized calcite micro-lattice on a curved PDMS matrix. The PDMS substrate, which immobilized the neck and RE parts of the calcite heterostructures, was initially attached on the curved tube surface. After the secondary mineralization and merging were done, the whole construct was detached and became rigid and freestanding. **d**, An optical micrograph (upper) and an SEM image (lower) of fragmented calcite sheets after intentional indentation, suggesting a merging of adjacent calcite heterostructures.

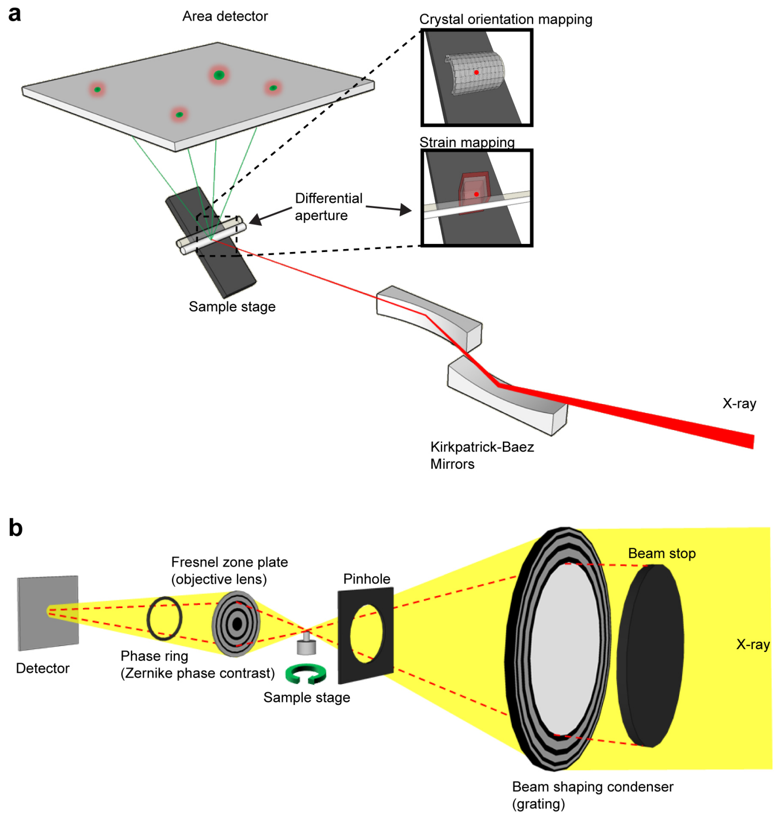

Supplementary Figure 12 | X-ray systems. a, Schematic of the X-ray Laue diffraction microscopy setup for crystal orientation mapping and strain analysis. **b**, Schematic illustrating transmission X-ray microscope used for tomography studies.

.

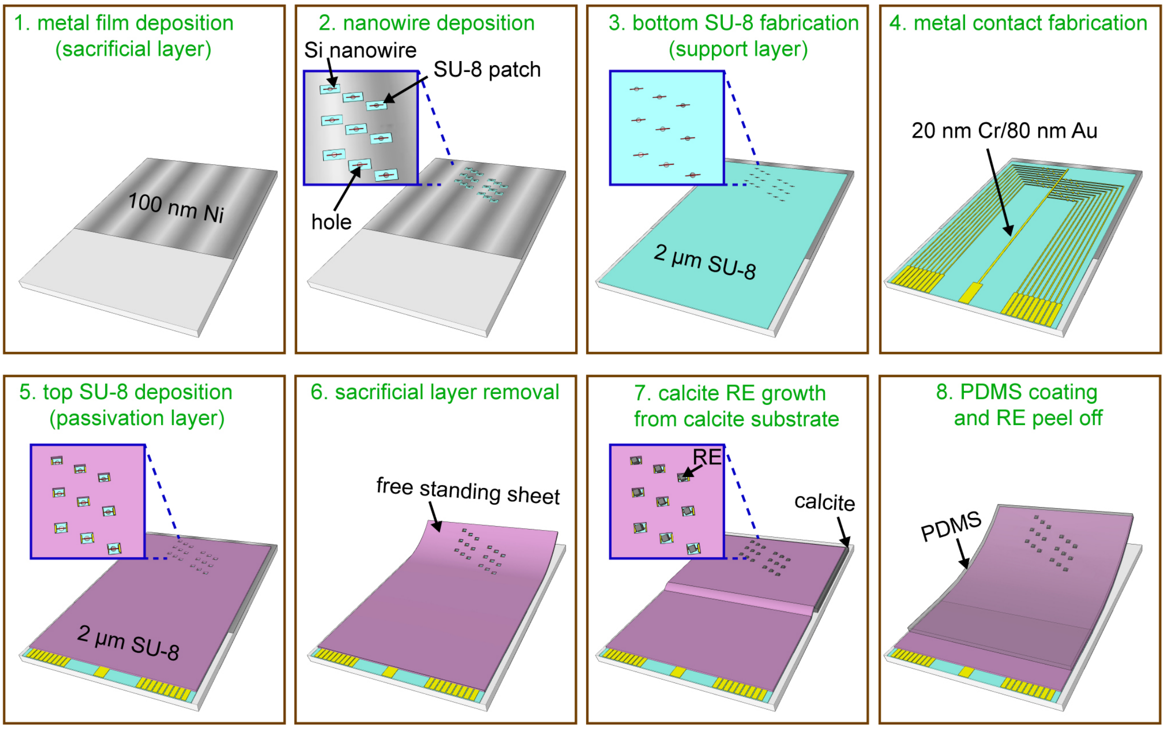

Supplementary Figure 13 | Schematic of the fabrication procedures for a flexible SiNW FET array with calcite-based switches.

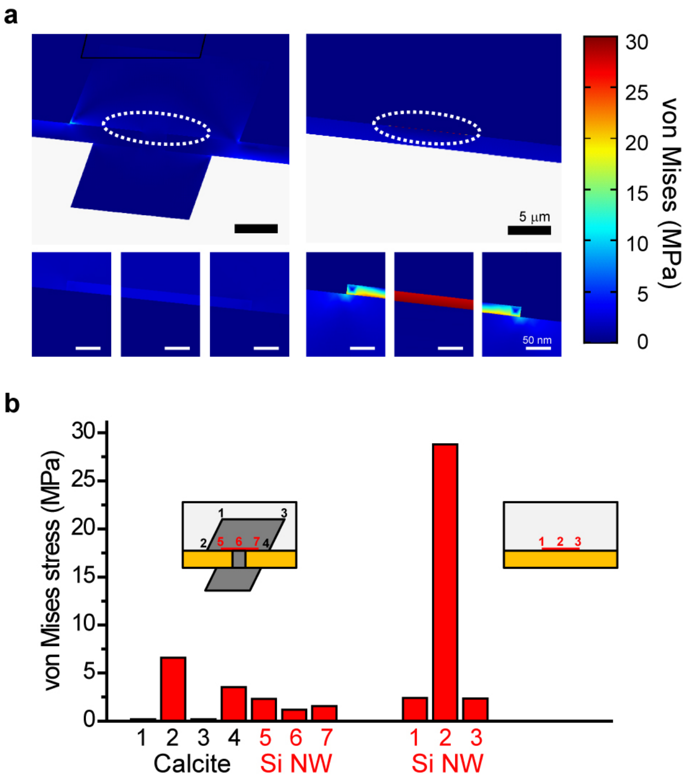

Supplementary Figure 14 | Finite element simulations showing von Mises stress of a Si nanowire with/without calcite encapsulation. Both stress distributions (**a**) and a bar chart (**b**) indicate that the rigid calcite heterostructure can significantly reduce the stress that is accumulated over the Si nanowire during bending of the substrate.

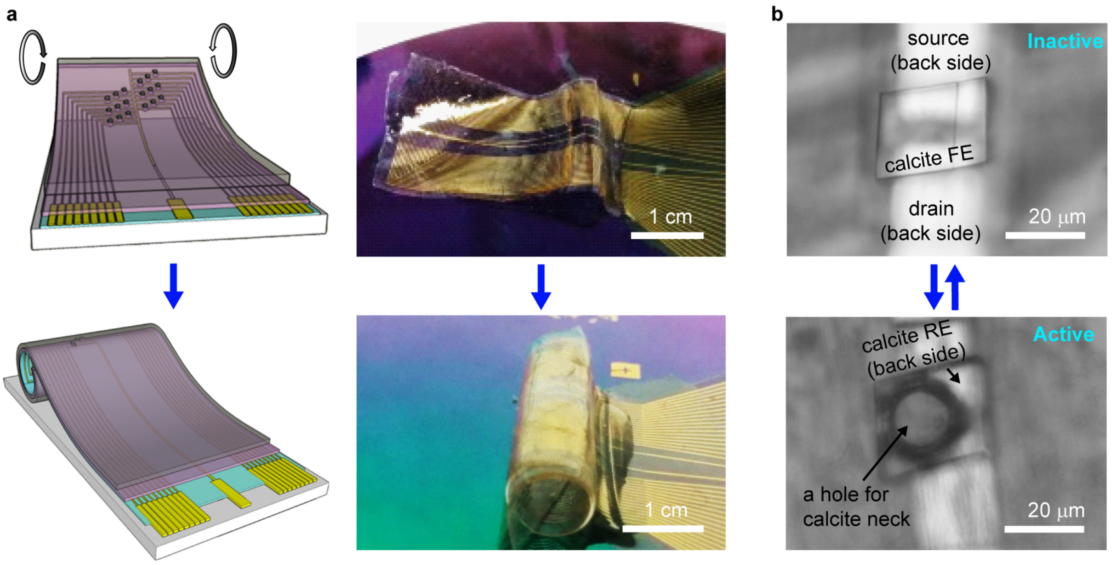

Supplementary Figure 15 | Calcite microarray for calcite encapsulation in flexible electronics. a, Schematics (left) and photographs (right) of flexible FET/calcite matrix before (upper) and after rolling up (lower). **b**, Optical micrographs of calcite/FET junctions at 'Inactive' (upper) and 'Active' (lower) Mode.

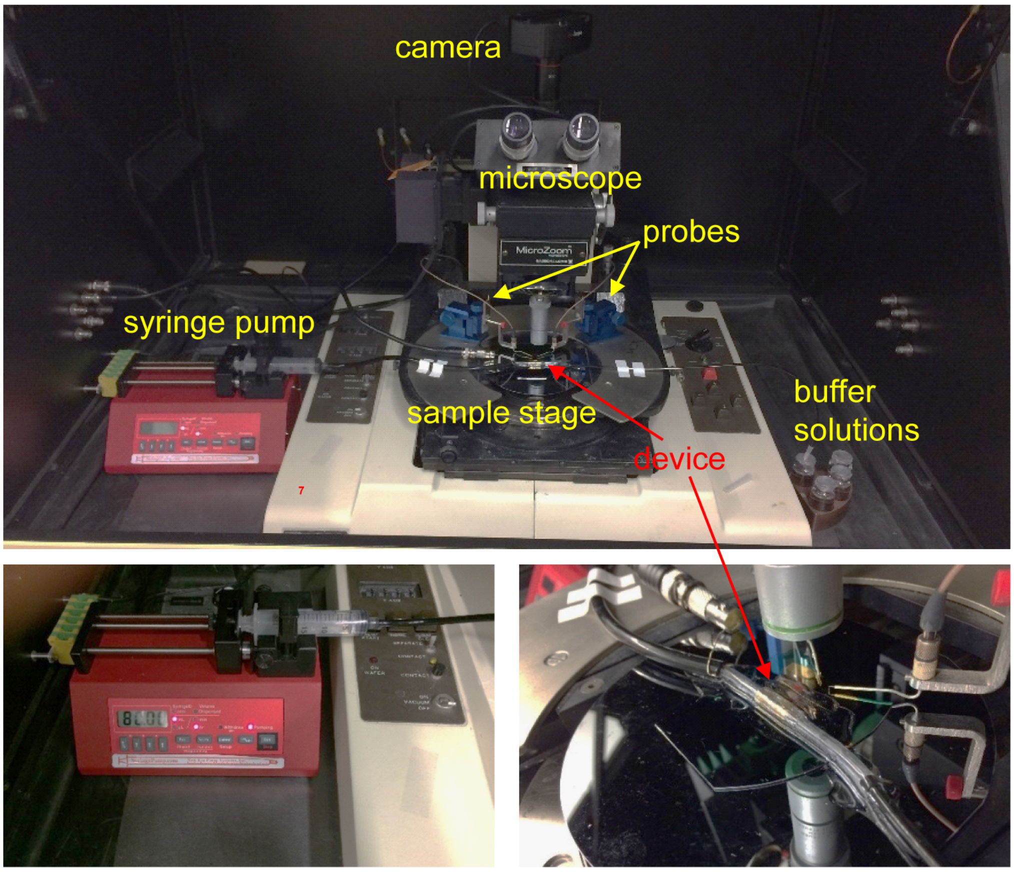

Supplementary Figure 16 | Measurement setup for the sensing experiment. The devices were mounted on a sample stage, and their electrical responses were recorded with a Keithley 2636A dual-channel source meter and with a reference electrode. The buffer solutions (including solutions for etching, regrowth and pH sensing) were supplied from the vials (right side) and eventually were collected into a syringe that was connected to a syringe pump (left side).

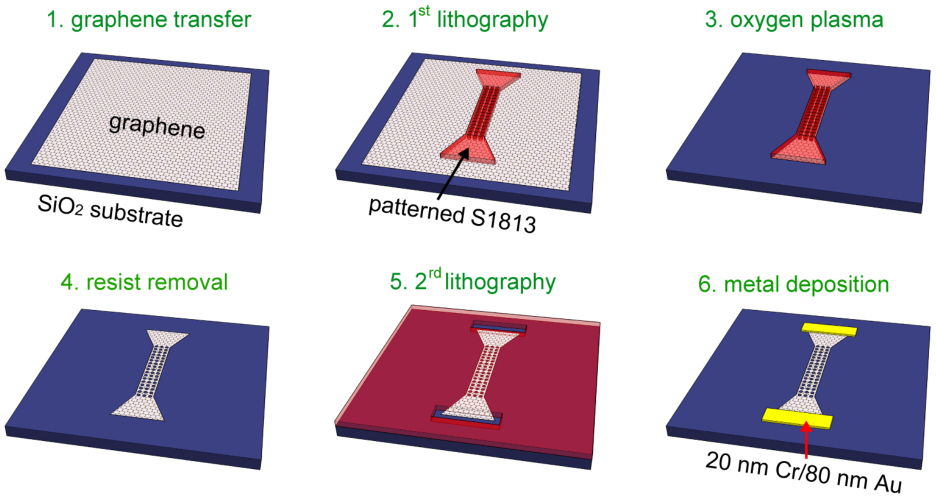

Supplementary Figure 17 | Schematic illustrating detailed fabrication steps of a graphene mesh-based FET. To prepare of graphene mesh-based devices, monolayer graphene was synthesized using a chemical vapour deposition method. Briefly, a copper foil (99.8%, 0.025 mm thick, Alfa Aesar) was placed at the center of a quartz tube reactor at a vacuum condition and heated to 1000 ºC. The copper foil was thermally annealed under the pressure of 800 mTorr with 35 sccm H_2 flow for 30 min. After the annealing process, 15 sccm CH_4 gas was introduced as a precursor for graphene growth and maintained for 15 min. The as-grown graphene layer on the copper foil was coated with a PMMA (PMMA, C4, MicroChem) supporting layer by spin coating (3000 rpm, 1 min). After immersion in 10 M ammonium persulfate (Sigma-Aldrich) solution for 4 h, the copper film was completely removed. The PMMA/graphene film was then carefully transferred onto fresh ultrapure water to remove the residual ammonium persulfate. We then applied 1st photolithography and O_2 plasma etching (100 W, 1 min) to define hole array with a diameter and the center-to-center distance of 3 μm and 6 μm, respectively. Through 2nd pattering step which including 2nd lithography, 20 nm Cr/ 80nm Au metal deposition and lift off, metal contact for the electrical measurement were obtained.

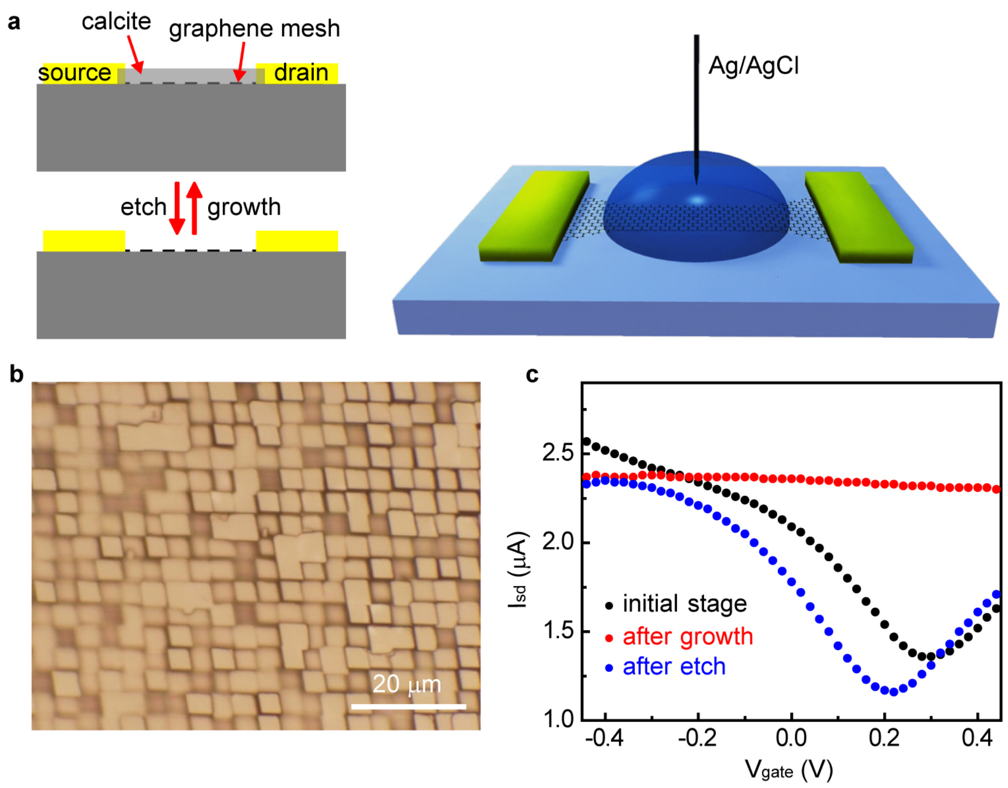

Supplementary Figure 18 | Graphene nanomesh devices. a, Schematics of switching operation via calcite growth or etching (left) and the water-gate experiment (right). **b**, Optical micrograph of mineralized calcite lattice over graphene mesh. **c**, Drain-source current versus water-gate voltage (I_{sd}–V_{gate}) curves, recorded at a drain-source bias of 0.1 V. The growth of calcite lattice can turn off the FET response, while subsequent etching can partially restore the initial FET sensitivity.

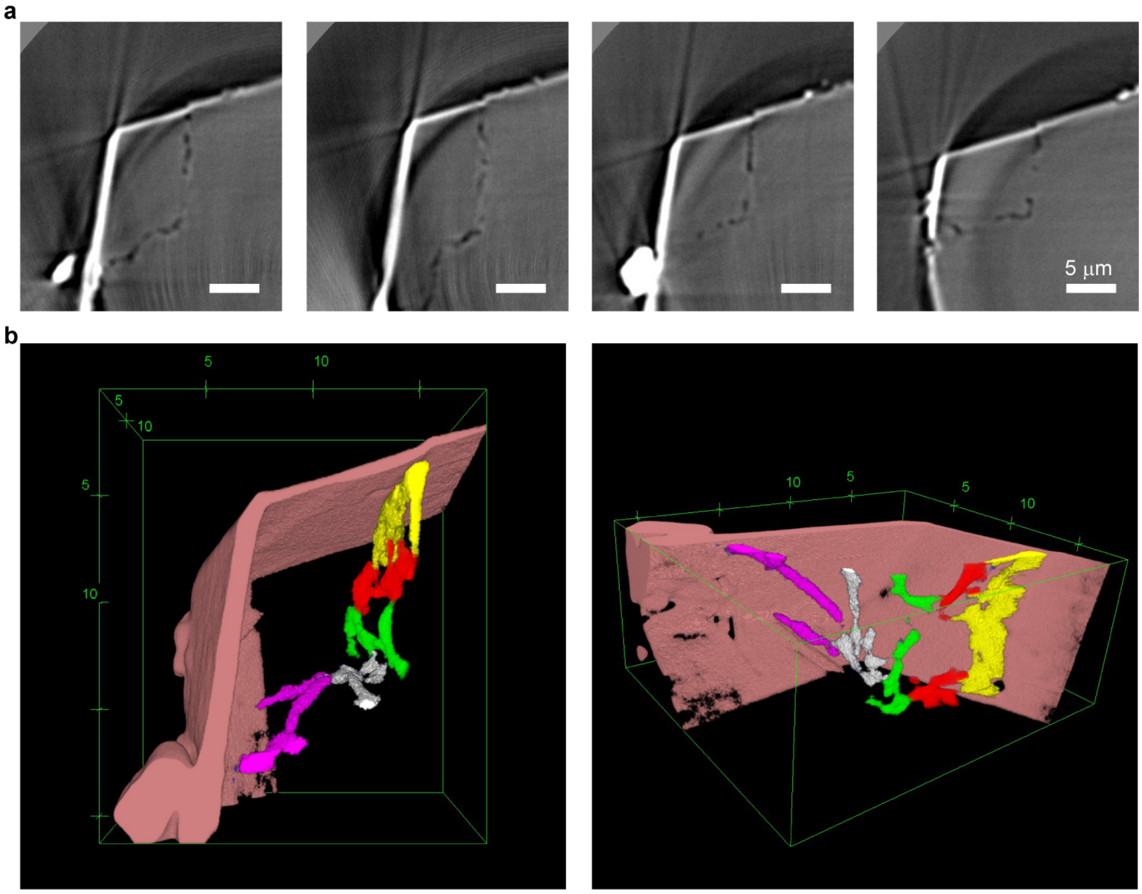

Supplementary Figure 19 | Collagen incorporation. a, Transmission X-ray microscopy sections of a calcite crystal with collagen incorporation. The holes inside calcite crystal suggest fibril inclusion, and they tend to accumulate on the $\{10\bar{1}4\}$ planes which gives parallelogram outlines in each section. **b**, 3D reconstructed transmission X-ray microscopy images with pseudo-colors showing exterior calcite crystal (peach) and incorporated collagen domains (yellow, red, green, white, purple).

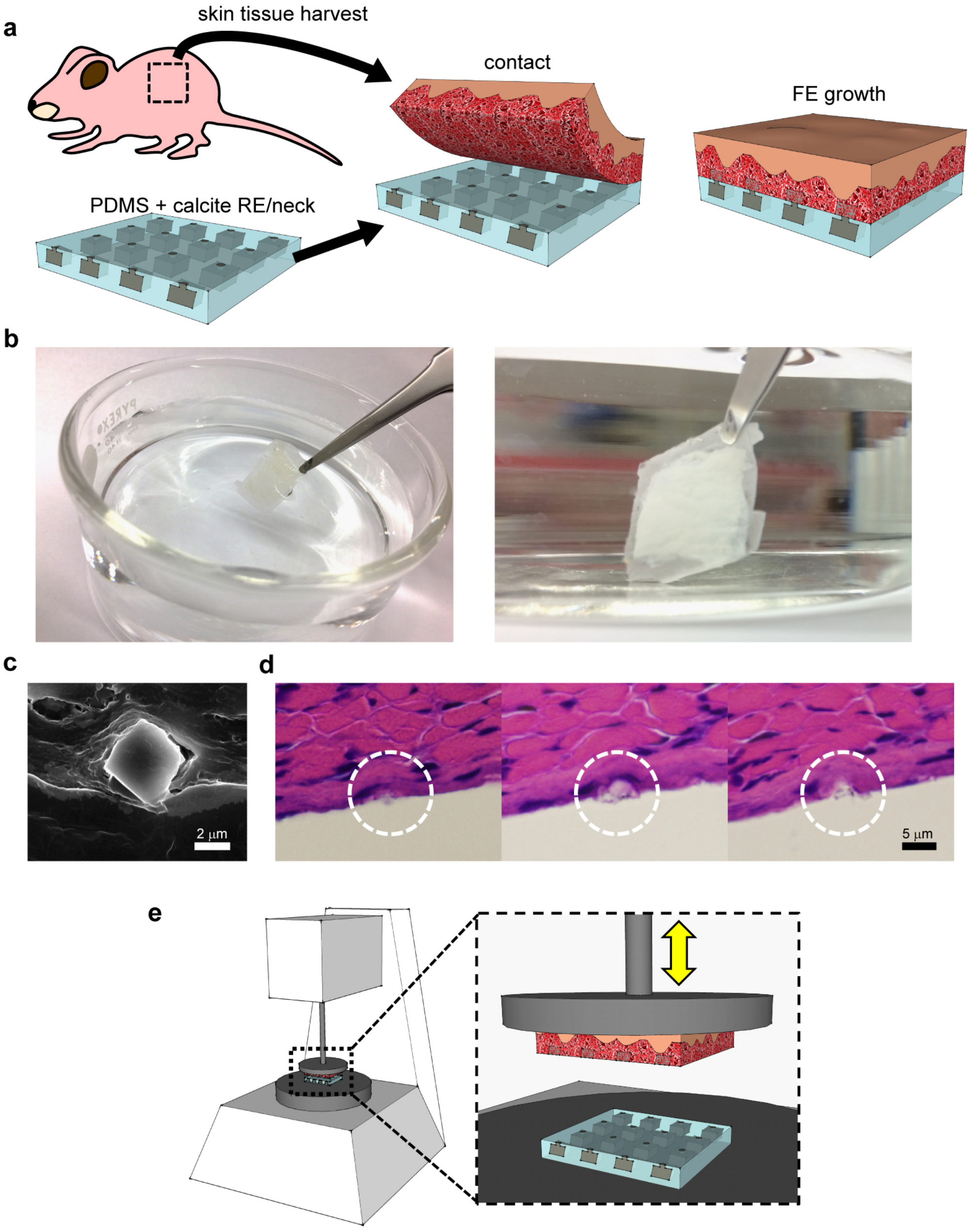

Supplementary Figure 20 | Inorganic focal adhesions. a, Schematics of a tissue adhesive with calcite-based inorganic focal adhesions. **b**, Photographs showing inorganic focal adhesion under water. **c**, An SEM image of the interface between neonatal rat dermis from excised skin tissue and a calcite-based adhesive, recorded after sectioning. **d**, A series of H&E stained 6 μm-thick sections, indicating the incorporation of extracellular matrix materials from the dermis in a single calcite crystal. **e**, A schematic of the rheometer setup for force-distance measurements of the skin/matrix samples.

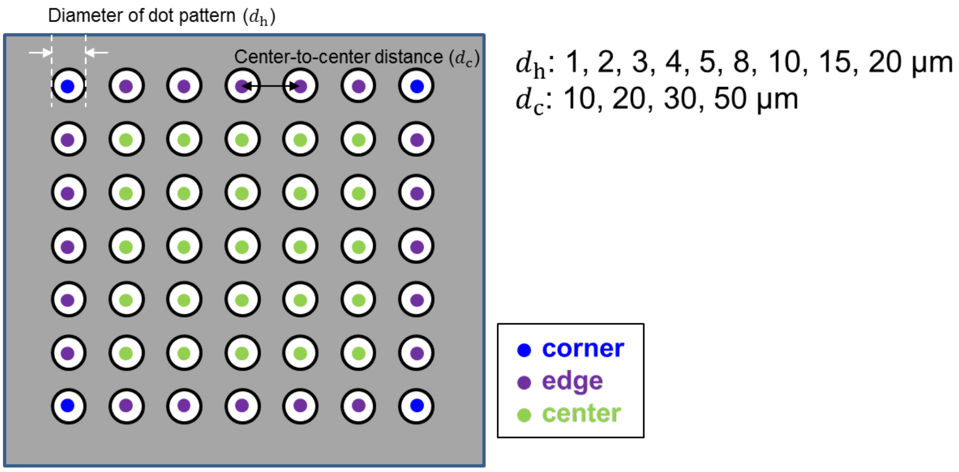

Supplementary Figure 21 | A design of 7×7 square array of hole patterns in the resist.

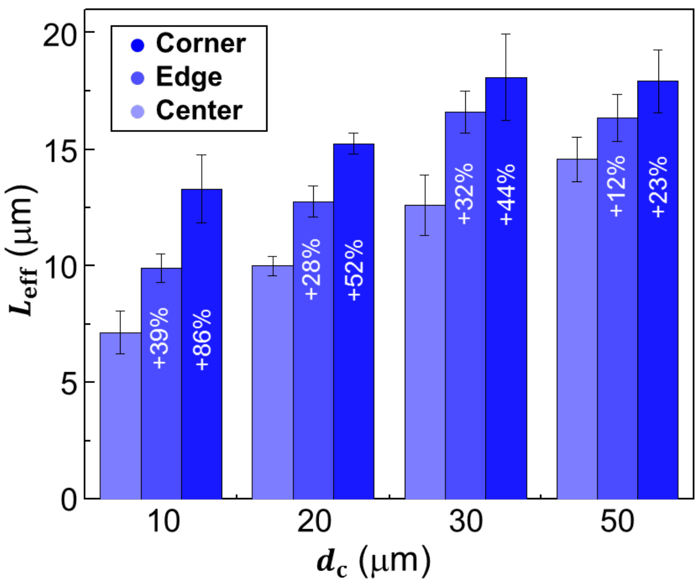

Supplementary Figure 22 | Location dependent effective lengths (L_{eff}) – center-to-center distances (d_c) plot. Error bar indicates standard deviation (n = 7).

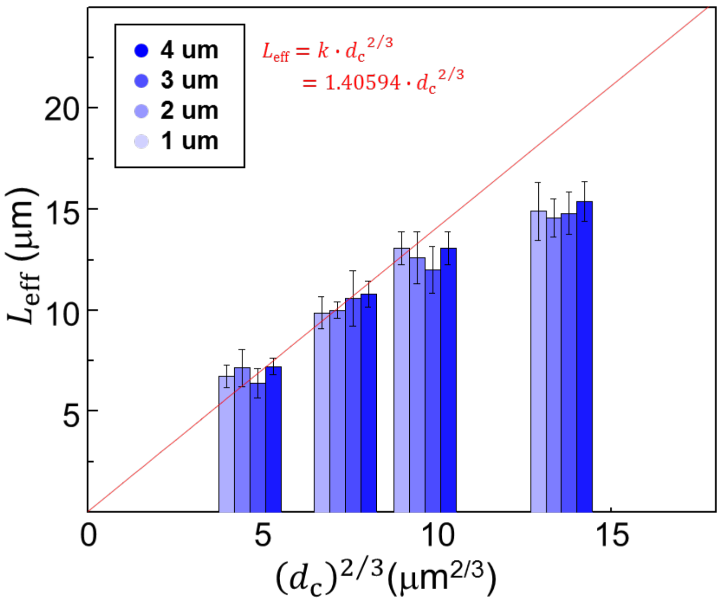

Supplementary Figure 23 | A plot of L_{eff} versus $(d_c)^{2/3}$ (blue column), and its theoretical limitation curve (red line) and formula. Error bar indicates standard deviation (n = 14).

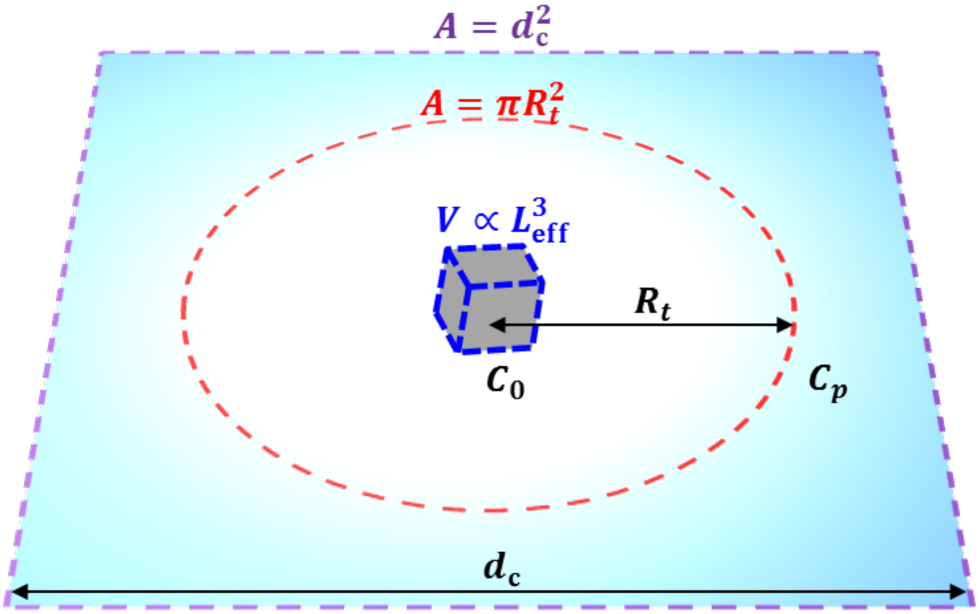

Supplementary Figure 24 | A schematic for the study of the growth kinetics. Consumption of adjacent ACC and vaterite particles during the calcite growth can yield a circular depletion zone.

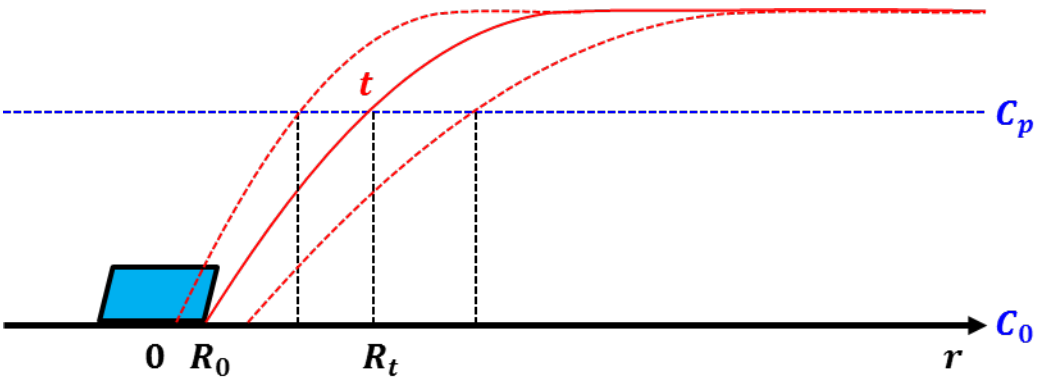

Supplementary Figure 25 | A schematic of time-dependent precursor concentration.

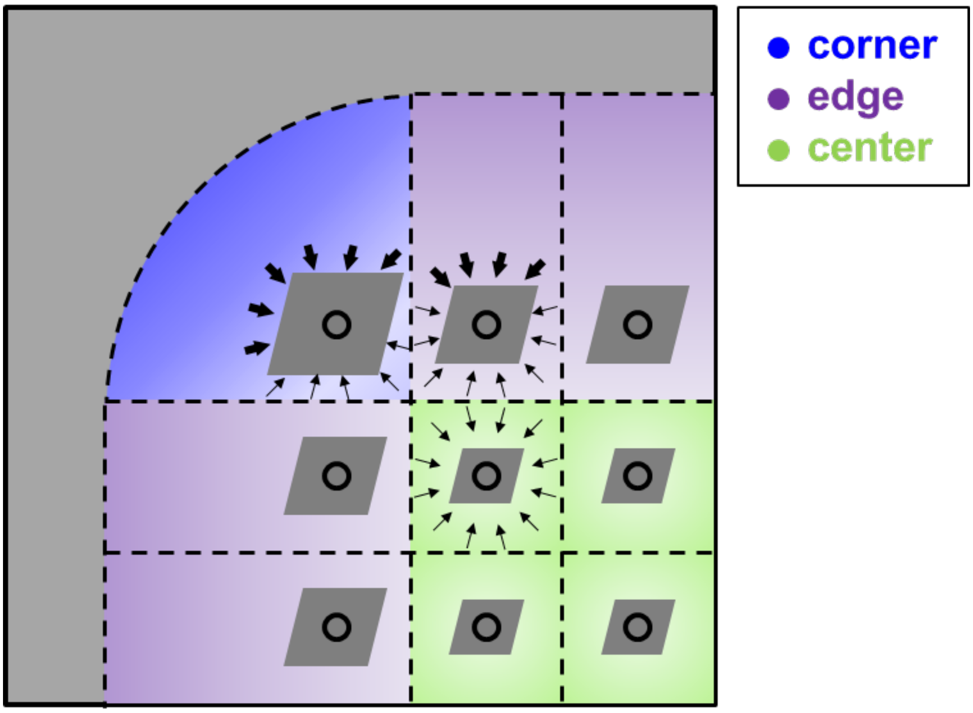

Supplementary Figure 26 | A schematic of 2D fluxes over the sample surface.

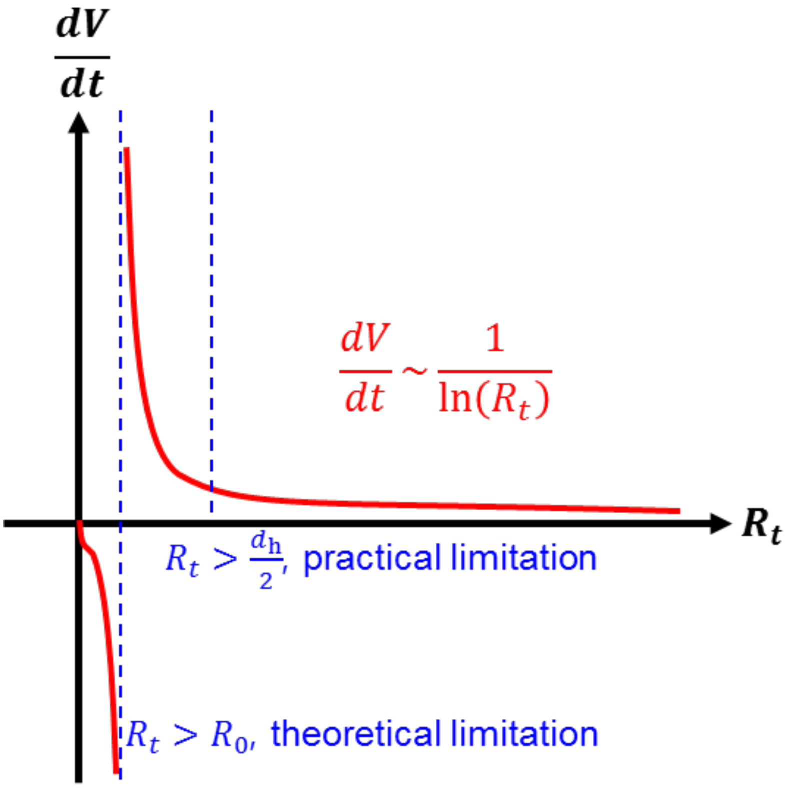

Supplementary Figure 27 | A predicted growth rate curve based on our model.

Supplementary Table 1. Selected materials and interfaces which can be used for underwater adhesives.

Research group	Interface materials	Target	Adhesion strength (N/m^2)	Work of adhesion (J/m^2)	Ref.
This paper	Calcite array	Skin (rat)	177,272* (SD=90,559)	158.70* (SD=120.19)	
Jeffrey M. Karp	PS-b-PAA	Skin (pig)	~9,300	~53.3	7
Phillip B. Messersmith	DOPA and PDMS	Si_3N_4	~149,200**	-	8
Jonathan J. Wilker	Poly(catechol-styrene)	Aluminum	~3,000,000	-	9
Julie C. Liu	Elastin-like polypeptide	Glass	~250,000	-	10
J. Herbert Waite	Polyelectrolyte complex	Glass	-	>2	11
	Catechol-functionalized poly(acrylic acid)	Mica	-	~0.05	12
Timothy K. Lu	DOPA and amyloid	Mica	-	~0.0209	13
Xuanhe Zhao	Functional silane over tough hydrogel	Glass	-	>1,000	14

*   N = 6. The surface area estimation only considers the calcite crystals.
** It was calculated based on an assumption that only the top surface of the pillars made contact with the AFM cantilever.

Supplementary References

1) Bots, P., Benning, L. G., Rodriguez-Blanco, J.-D., Roncal-Herrero, T. & Shaw, S. Mechanistic Insights into the Crystallization of Amorphous Calcium Carbonate (ACC). *Cryst. Growth Des.* **12**, 3806-3814 (2012).

2) Radha, A. V., Forbes, T. Z., Killian, C. E., Gilbert, P. U. P. A. & Navrotsky, A. Transformation and crystallization energetics of synthetic and biogenic amorphous calcium carbonate. *Proc. Natl. Acad. Sci.* **107**, 16438-16443 (2010).

3) Plummer, L. N. & Busenberg, E. The solubilities of calcite, aragonite and vaterite in CO_2-H_2O solutions between 0 and 90°C, and an evaluation of the aqueous model for the system $CaCO_3$-CO_2-H_2O. *Geochim. Cosmochim. Acta* **46**, 1011-1040 (1982).

4) Brečević, L. & Nielsen, A. E. Solubility of amorphous calcium carbonate. *J. Cryst. Growth* **98**, 504-510 (1989).

5) Rodriguez-Blanco, J. D., Shaw, S. & Benning, L. G. The kinetics and mechanisms of amorphous calcium carbonate (ACC) crystallization to calcite, viavaterite. *Nanoscale* **3**, 265-271 (2011).

6) Aizenberg, J., Black, A. J. & Whitesides, G. M. Control of crystal nucleation by patterned self-assembled monolayers. *Nature* **398**, 495-498 (1999).

7) Yang, S. Y., O'Cearbhaill, E. D., Sisk, G. C., Park, K. M., Cho, W. K., Villiger, M., Bouma, B. E., Pomahac, B. & Karp, J. M. A bio-inspired swellable microneedle adhesive for mechanical interlocking with tissue. *Nat. Commun.* **4**, 1702 (2013).

8) Lee, H., Lee, B. P. & Messersmith, P. B. A reversible wet/dry adhesive inspired by mussels and geckos. *Nature* **448**, 338-341 (2007).

9) North, M. A., Del Grosso, C. A. & Wilker, J. J. High Strength Underwater Bonding with Polymer Mimics of Mussel Adhesive Proteins. *ACS Appl. Mater. Interf.* **9**, 7866-7872 (2017).

10) Brennan, M. J., Kilbride, B. F., Wilker, J. J. & Liu, J. C. A bioinspired elastin-based protein for a cytocompatible underwater adhesive. *Biomaterials* **124**, 116-125 (2017).

11) Zhao, Q., Lee, D. W., Ahn, B. K., Seo, S., Kaufman, Y., Israelachvili, J. N. & Waite, J. H. Underwater contact adhesion and microarchitecture in polyelectrolyte complexes actuated by solvent exchange. *Nat. Mater.* **15**, 407-412 (2016).

12) Ahn, B. K., Das, S., Linstadt, R., Kaufman, Y., Martinez-Rodriguez, N. R., Mirshafian, R., Kesselman, E., Talmon, Y., Lipshutz, B. H., Israelachvili, J. N. & Waite, J. H. High-performance mussel-inspired adhesives of reduced complexity. *Nat. Commun.* **6**, 8663 (2015).

13) Zhong, C., Gurry, T., Cheng, A. A., Downey, J., Deng, Z., Stultz, C. M. & Lu, T. K. Strong underwater adhesives made by self-assembling multi-protein nanofibres. *Nat. Nano.* **9**, 858-866 (2014).

14) Yuk, H., Zhang, T., Lin, S., Parada, G. A. & Zhao, X. Tough bonding of hydrogels to diverse non-porous surfaces. *Nat. Mater.* **15**, 190-196 (2016).
